# secDrug: a pipeline to discover novel drug combinations to kill drug-resistant multiple myeloma cells using a greedy set cover algorithm and single-cell multi-omics

**DOI:** 10.1038/s41408-022-00636-2

**Published:** 2022-03-09

**Authors:** Harish Kumar, Suman Mazumder, Sayak Chakravarti, Neeraj Sharma, Ujjal Kumar Mukherjee, Shaji Kumar, Linda B Baughn, Brian G Van Ness, Amit Kumar Mitra

**Affiliations:** 1 Department of Drug Discovery and Development, Harrison College of Pharmacy, Auburn University, Auburn, AL USA; 2 Center for Pharmacogenomics and Single-Cell Omics initiative (AUPharmGx), Harrison College of Pharmacy, Auburn University, Auburn, AL USA; 3 Division of Laboratory Genetics, Department of Laboratory Medicine and Pathology, Mayo Clinic, Rochester, MN USA; 4 Department of Business Administration, University of Illinois at Urbana-Champaign, Champaign, IL USA; 5 Carle Illinois College of Medicine, University of Illinois at Urbana-Champaign, Champaign, IL USA; 6 Division of Hematology, Department of Internal Medicine, Mayo Clinic, Rochester, MN USA; 7 Department of Genetics, Cell Biology & Development, University of Minnesota, Minneapolis, MN USA

**Keywords:** Translational research, Myeloma, Myeloma, Drug development

## Abstract

Multiple myeloma, the second-most common hematopoietic malignancy in the United States, still remains an incurable disease with dose-limiting toxicities and resistance to primary drugs like proteasome inhibitors (PIs) and Immunomodulatory drugs (IMiDs).

We have created a computational pipeline that uses pharmacogenomics data-driven optimization-regularization/greedy algorithm to predict novel drugs (“secDrugs”) against drug-resistant myeloma. Next, we used single-cell RNA sequencing (scRNAseq) as a screening tool to predict top combination candidates based on the enrichment of target genes. For in vitro validation of secDrugs, we used a panel of human myeloma cell lines representing drug-sensitive, innate/refractory, and acquired/relapsed PI- and IMiD resistance. Next, we performed single-cell proteomics (CyTOF or Cytometry time of flight) in patient-derived bone marrow cells (ex vivo), genome-wide transcriptome analysis (bulk RNA sequencing), and functional assays like CRISPR-based gene editing to explore molecular pathways underlying secDrug efficacy and drug synergy. Finally, we developed a universally applicable R-software package for predicting novel secondary therapies in chemotherapy-resistant cancers that outputs a list of the top drug combination candidates with rank and confidence scores.

Thus, using 17AAG (HSP90 inhibitor) + FK866 (NAMPT inhibitor) as proof of principle secDrugs, we established a novel pipeline to introduce several new therapeutic options for the management of PI and IMiD-resistant myeloma.

## Introduction

Multiple myeloma (MM) is the second-most common hematopoietic malignancy in the United States [1]. MM is an age-dependent plasma cell neoplasm characterized by clonal expansion of malignant antibody-producing post-germinal- center B cell-derived plasma cells within the bone marrow with significant complexity and heterogeneity at the molecular level [1]. Proteasome inhibitors (PIs) are standard-of-care chemotherapeutic agents for myeloma that impede tumor metastasis and angiogenesis by accelerating unfolded protein response (UPR) or the ubiquitin-dependent proteolysis of important regulatory proteins involved in key physiological and pathophysiological cellular processes in cancer cells and by interfering with the NF-κB-enabled regulation of cell adhesion-mediated drug resistance [2–6]. Bortezomib (Bz/Velcade) was the first PI to be approved by U.S. Food and Drug Administration (FDA) for clinical application in 2003 for the treatment of relapsed and refractory myeloma [1, 7, 8]. Other examples include second-generation PIs Carfilzomib (Cz/Kyprolis) and the oral medication Ixazomib (Ix /Ninlaro/MLN9708) [7–9]. PIs are effective anti-MM drugs when used alone or in combination with other anti-cancer agents like immunomodulatory drugs (IMiDs), alkylating agents, topoisomerase inhibitors, corticosteroids, and histone deacetylase inhibitors (HDACis) [1, 3]. However, despite these and other recent improvements in therapies, myeloma still remains a difficult-to-cure disease with dose-limiting toxicities and drug resistance and a median survival rate of only around 7 years [7, 10]. Not all patients respond equally well to treatment and those who do often develop resistance over the course of treatment. Drug resistance may therefore be categorized into (1) innate resistance already present in drug-naive patients who never respond to treatment, or (2) emerging/acquired resistance where a patient’s tumor ultimately undergoes relapse or “acquires” the ability to resist therapy in the course of treatment despite good response to initial treatment [2, 11]. Therefore, there is an urgent need to search for novel secondary therapeutic options where new agents may be combined with standard-of-care drugs to achieve synergistic effects for treating drug resistance in myeloma.

Deciphering key features within patients underlying tumor heterogeneity and personalized sensitivity to chemotherapy is essential to predict the efficacy of anti-cancer drugs and to prevent delay in the selection of more effective alternative strategies [2, 11–14]. However, assessing the survival endpoints in clinical applications requires the treatment of a large number of patients with these drugs that need to be measured in months to years. Therefore, developing prediction algorithms of response can be a long process. One alternative is to use collections of human cancer cell lines from patient tumors that represent a broad spectrum of the biological and genetic heterogeneity of cancer, commonly known as in vitro modeling of drug response. We have compiled a panel of >70 human myeloma cell lines (HMCLs) representing the broad spectrum of biological and genetic heterogeneity of myeloma patients [15].

In this study, we have developed a computational method called secDrug for discovering novel synergistic secondary drug combinations that may effectively reverse resistance as combination regimens and allow for reduced dosing and toxicity of FDA-approved myeloma drugs. Next, we introduced single-cell transcriptomics as a novel screening tool for prioritizing secDrug combinations based on the subclonal expression of the drug targets and observed that the 17AAG + FK866 combination is potentially highly efficacious.

Further, to validate our prediction results, we used our HMCL panel as in vitro model system representing inter-individual heterogeneity in drug-response/resistance to show that the top predicted secondary secDrugs are indeed effective against PI- and IMiD resistance as single agents or as a combination. Further, using 17-AAG (an HSP90 inhibitor) as the test secDrug, we added functional assays, next-generation RNA sequencing, CRISPR-based gene editing, and high-dimensional mass cytometry (CyTOF/cytometry time of flight) in primary bone marrow cells (PMCs; ex vivo model system) from myeloma patients to create a multi-pronged approach/pipeline to discover, validate and characterize novel drugs as potential secondary choices for circumventing resistance to primary drugs in myeloma and to generate better treatment outcomes. This also allowed the identification of differentially expressed (DE) genes and novel pathways associated with the successful drug combinations.

## Materials and methods

### In silico prediction of secondary drugs

Design and development of the secDrug pipeline are non-trivial and mathematically involved (details provided in Supplementary Methods section). Briefly, we utilized we used the vast array of human cancer cell lines in the Genomics of Drug Sensitivity in Cancer (GDSC version GDSC1000) database and created a pharmacogenomics data-driven greedy algorithm-based set-covering computational optimization method followed by a regularization technique to seek all secondary drugs that could kill the maximum number of cell lines of the test disease (B-cell cancers) resistant to the test drug (PI) in a sequential manner ordered by the number of cell lines killed. A greedy algorithm constructs a solution to an *optimization problem* piece by piece through a sequence of choices to find the overall, or globally, optimal solution. The GDSC1000 database is the largest public collection of information on sensitivity to >250 drugs covering a wide range of targets and processes involved in cancer biology in >1000 human cancer cell lines [16, 17]

### Drugs, reagents, antibodies, and kits

Ixazomib (Ixa) was procured from Takeda (Takeda Pharmaceuticals Inc., Deerfield, IL, USA). All other drugs were purchased from Selleck Chemicals. Drugs were dissolved in dimethyl sulfoxide (DMSO) and stored at −20 °C. Recombinant Human IL-6 was obtained from Peprotech.

Cleaved caspase-3/8/9, HSP90, c-Myc, p65, and IRF4 antibodies were purchased from Cell signaling Technology (CST). Monoclonal Anti-β-Actin-Peroxidase antibody produced in mouse was purchased from Sigma-Aldrich (St Louis, MO). Goat anti-Mouse/Rabbit IgG (H + L) secondary antibody (HRP conjugated) was obtained from Thermo Fisher Scientific. DHE (Dihydroethidium) assay kit and JC-1 Mitochondrial Membrane Potential (MMP) assay kit were purchased from Abcam (Boston, MA). Caspase-Glo 3/7 Assay System and CellTiter-Glo 2.0 Assay were purchased from Promega (Madison, WI).

### Human myeloma cell lines (HMCLs)

HMCLs generated through the immortalization of primary myeloma cells were used as in vitro model systems to screen top secDrugs against sensitive, innate resistant, and acquired (Parental/P vs. clonally-derived resistant/R pairs generated using dose escalation over a period of time) myeloma [15]. We have also generated in vitro drug response profile for the four PIs: Bz, Cz, Oprozomib (Opz), and Ix as single agents in all the HMCLs included in the panel. PI-sensitivity in these cell lines was highly correlated, which suggests that any of these four PIs could be used as surrogates [15]. Therefore, we used Ixazomib as the representative PI in this study. Further, we have used machine learning-based computational approaches to derive a gene expression signature predictive of baseline PI-response in myeloma [15]. The creation of the ANBL6 N-*ras* (ANBL6/Ras) codon 61 activating mutant cell line has been described earlier [18]. The IMiD resistant cell line, MM1S LenR, was obtained as a gift from Dr. Keith Stewart, Mayo Clinic, AZ. All cell lines were authenticated at source and tested randomly at regular intervals at the AU Center for Pharmacogenomics and Single-Cell Omics (AUPharmGx) using GenePrint 24 System (Promega). All cell lines are mycoplasma negative. HMCLs were maintained in HMCL media supplemented with IL-6 [19].

### Human primary myeloma cells (PMCs)

Bone marrow-derived CD138+ ve patient PMCs were obtained through Mayo Clinic, MN following written informed consent and used as ex vivo model systems. Prior IRB approval was obtained from the Mayo Clinic review board. Participants were identified by number, not by name.

### Establishment of RPMI8226 Hsp90 CRISPR-knockout cell line

Chemically-modified synthetic single-guide RNA (sgRNA) were designed targeting the Hsp90AA1 gene and synthesized by Synthego (Synthego Corporation, Menlo Park, CA). The sgRNAs were required to meet strict off-target requirements of at least 2 mismatches within an early exon and target a common exon present in the majority of annotated transcripts. The sgRNAs were complexed together with the spCas9 to form a ribonucleoprotein (RNP). The RNPs were then delivered to RPMI8226 cells via an optimized electroporation setting. The transfected cells were then recovered for 2 days before the edits created were evaluated. Positive control sgRNA (RELA) were transfected at the same time. The edited site was PCR-amplified, and Sanger sequencing was performed on the amplicons Sequencing data was then analyzed using Synthego’s Inference of CRISPR Edits (ICE) software tool to determine the percentage of knock-out (KO) sequences of the genetic target [20]. ICE identifies the editing frequency and the specific indels present in the pool. Additionally, ICE calculates the frequency of the desired KO, reported as the KO score. Finally, once minimum KO editing efficiency was confirmed, RPMI8226/Hsp90KO cells were expanded and QC tested.

### In vitro chemosensitivity assays and drug synergy analysis

Cells were treated with increasing concentrations of secDrugs and PIs (represented by Ixazomib) or IMiDs (represented by Lenalidomide) as single agents or in combination for 48 h, and cytotoxicity assays were performed using CellTiter-Glo^®^ Luminescent cell viability assay (Promega Madison, WI). Luminescence was recorded in a Neo2 Microplate Reader (Biotek), and half-maximal inhibitory concentration (IC_50_) values were determined using GraphPad Prism software by calculating the nonlinear regression using sigmoidal dose-response equation (variable slope). Drug synergy was calculated using Calcusyn software based on Chou–Talalay’s combination index (CI) method and the isobologram algorithm (Biosoft, US) [21].

### Apoptosis assays

Caspase-3/7 activity assay was performed on the HMCLs using Caspase-Glo 3/7 luminescent assay kit according to manufacturer’s instructions (Promega Madison, WI) using Neo 2 Microplate Reader (Biotek). Cell death by apoptosis was also measured by immunoblotting analysis.

### Determination of superoxide levels

Cells were incubated with 5 μM DHE (in RPMI) for 15 min in the dark at 37 °C. Cells were then washed once with cell-based assay buffer, and red fluorescence was recorded by Synergy Neo2 multi-plate reader.

### Measurement of mitochondrial membrane potential (MMP)

Cells were incubated with 5 μM JC-1 dye for 15 min in the dark at 37 °C and washed twice in PBS, and then analyzed for red and green fluorescence by Synergy Neo2 multi-plate reader.

### Mass cytometry (CyTOF)

Thirty-seven antibody targets directed against cell surface and intracellular markers were utilized as Immunphenotyping Panel for CyTOF analysis. The Antibody markers and respective metal conjugate are described in Supplementary Table S1. Panels were designed using the web-based panel designer software: Maxpar Panel Designer (www.fluidigm.com) for optimal signals, minimum background due to oxidation, isotopic purity, and sufficient sensitivity for each targeted marker. Prelabeled antibodies were purchased from Fluidigm. Purified antibodies from Biolegend and Santacruz Biotechnology were labeled using an X8 polymer MaxPAR antibody conjugation kit (Fluidigm) according to the manufacturer’s instructions. CyTOF analysis was performed on PMCs treated with DMSO (vehicle/control), 0.2 µM 17AAG,1 µM 17AAG, and 5 µM 17AAG.

### CyTOF data analysis

Cytobank software version 7.3.0 (Santa Clara, CA) was used for cleanup of cell debris, removal of doublets and dead cells. Cleaned fcs files were further gated and analyzed by Cytobank. Plasma cells were identified as CD19−, CD16−, CD3−, CD38+, and kappa OR lambda+ (based on each patient’s kappa or lambda restriction from clinical flow data). If the plasma cells had diminished surface CD38 expression as a result of previous daratumumab exposure, CD229 was used as a positive selection marker. T-distributed stochastic neighbor embedding (t-SNE), viSNE, and FlowSom plots were generated to visualize the subpopulation architecture based on markers of interest. Relative marker intensities and cluster abundances per sample were visualized by a heatmap.

### Single-cell RNA sequencing (scRNAseq)

Automated single-cell capture and cDNA synthesis were performed at ~1500 tumor cells/sample using 10× Genomics Chromium platform that uses droplet-sequencing-based chemistry. Single-cell RNA sequencing was performed on Illumina HiSeq 2500 NGS platform (Paired-end. 2 × 125 bp, 100 cycles. v3 chemistry) at >50 million reads per sample.

### scRNAseq data analysis

scRNAseq datasets were obtained as matrices in the Hierarchical Data Format (HDF5 or H5). A combination of Seurat and Partek Flow software packages was used to pre-process the data and perform single-cell transcriptomics analysis. Highly variable genes for clustering analysis were selected based on a graph-based clustering approach. The visualization of cell populations was performed by t-SNE.

### Next-generation RNA sequencing (NGS)

HMCLs were plated at a density of 4 × 10^5^ cells per mL, and 0.5 μM of 17-AAG was added as a single agent or in combination with 15 nM of Ixazomib. Baseline (untreated) and post-treatment (treated) cells were collected 24 h post-treatment. High-quality RNA was extracted using QIAshredder and RNeasy kit (Qiagen). RNA concentration and integrity were assessed using Nanodrop-8000 and Agilent 2100 Bioanalyzer and stored at −80 °C. An RNA integrity number threshold of eight was applied, and RNA-seq libraries were constructed using Illumina TruSeq RNA Sample Preparation kit v2.

NGS Libraries were size-selected, and RNA sequencing (RNAseq) was performed on Illumina’s NovaSeq platform using 150 bp paired-end protocol with a depth of >20million reads per sample.

### RNAseq data analysis

Gene expression data were pre-processed, log_2_-transformed, and analyzed using a combination of command-line based analysis pipeline (DEseq2 and edgeR) and Partek Flow software to identify differential gene expression profiling (GEP) signatures. Genes with mean counts<10 were removed, and CPM (counts per million) data was used to perform differential expression testing to identify GEP signatures. Due to the small sample sizes, we used GSA to perform differential gene expression analysis between groups that applies limma, an empirical Bayesian method, to detect the DE genes (DEGs). Genes with mean fold-change > |1| and *p* < 0.05 were considered as the threshold for reporting significant differential gene expression. Heatmaps were generated using unsupervised hierarchical clustering (HC) analysis based on the top DEGs.

### Pathway analysis

Ingenuity pathway analysis (IPA) software (QIAGEN) was used to identify the molecular pathways and upstream regulators predicted to be activated or inhibited in response to 17-AAG treatment (single-agent and combination with PIs) based on the list of significantly differentially regulated genes [22].

### Western Blotting

HMCLs treated with 17-AAG alone, Ixa alone, or 17-AAG + Ixa combination were harvested, washed, and lysed using radioimmunoprecipitation assay (RIPA) lysis buffer containing 50 mM Tris-HCl, pH 7.5, 150 mM NaCl, 1% NP40, 5 mM EDTA, 1 mM DTT, phosphatase, and protease inhibitors cocktail (Sigma) and incubated on ice for 15 min. Samples were then centrifuged at 14,000 rpm at 4 °C for 30 min. The supernatant was then aspirated and quantified using Pierce^™^ BCA Protein Assay Kit (Thermo Scientific). Samples were solubilized in sodium dodecyl sulfate-polyacrylamide gel electrophoresis sample buffer, and equal amounts of protein were loaded per lane of 10% sodium dodecyl sulfate-polyacrylamide gels and transferred onto PVDF membranes (Millipore; Billerica, MA). Membranes were blocked in TBS with SuperBlock^™^ blocking buffer (Thermo Fisher), incubated with primary antibodies and secondary antibodies in TBS with 0.2% Tween 20 and 2.5% bovine serum albumin. Immunoreactivity was detected by chemiluminescent HRP substrate (Bio-Rad), and the exposed image was captured using a ChemiDoc^™^ MP Imaging System (Bio-Rad). Densitometry analysis was performed using Image J software.

### Statistical analysis

All statistical analyses were performed using R (the project for statistical computing and graphics) and GraphPad Prism 9.0 software. All tests were two-sided, and *p* < 0.05 was considered statistically significant.

## Results

### Identification of secondary drugs using secDrug algorithm

Details on the design and development of the secDrug pipeline are provided in the Supplementary Methods section. Briefly, a novel modified greedy algorithm-based set-covering computational optimization-regularization pipeline was used to identify all secondary drugs that could kill the maximum number of cell lines in the GDSC1000 database belonging to the test disease (B-cell cancers including myeloma) and which are resistant to the PI/PI drug Bortezomib (Bz/Velcade; the primary anti-myeloma drug). A total of 1091 cells lines were present in the GDSC1000 database [16, 17]. The following filtering criteria were applied to select computable B-cell lines: target cell—B-cell; cancer type—blood; tissue—blood; histology—lymphoid_neoplasm/haematopoietic_neoplasm; site—haematopoietic_and_lymphoid_tissue; no missing data). A total of 94 cell lines satisfied the above filtering criteria and were selected for further analysis. IC_50_ values were processed, imputed, and categorized as S (PI-sensitive), R (PI-resistant), and N (“Neutral”/Intermediate PI IC_50_ values) prior to analysis. We applied our computation algorithm to the GDSC1000 dataset and predicted the top secDrugs that can be best combined with PIs to achieve response in N and R lines. The predicted top secondary drug combinations in PI-resistant + PI-neutral B-cell cancers with a PI backbone are shown in Table 1. These include HSP90 inhibitor (17-AAG), Nicotinamide phosphoribosyltransferase or Nampt inhibitor (FK866), PIKfyve inhibitor (YM201636), Raf inhibitor (PLX-4720), Bcl2 inhibitor (Navitoclax), SB505124 (transforming growth factor-β type I receptor, ALK4, ALK7 inhibitor), S6K1-specific inhibitor (PF-4708671), and the neddylation inhibitor (MLN4924). Furthermore, when only the top PI-resistant cell lines (R; highest 33% PI IC_50_) were considered, the following drugs were predicted to be highly effective: 17.AAG, PLX4720, YM201636, and the AKT inhibitor KIN001.102.

**Table 1:** Detailed list of top combination treatment regimens with a proteasome inhibitor (PI) backbone predicted using the secDrug computational algorithm.

Sl. No.	NoDrug	PI only (%)	PI + 2 secDrugs	PI + 3 secDrugs
1			PI + FK866 + 17.AAG	PI + FK866 + 17.AAG + SB216763
	0	33.0	72.2%	82.5%
2			PI + XAV939 + 17.AAG	PI + XAV939 + 17.AAG + VNLG.124
	0	33.0	71.1%	83.5%
3			PI + PF.4708671 + Bleomycin	PI + PF.4708671 + Bleomycin + FK866
	0	33.0	76.3%	87.6%
4			PI + Bleomycin + SB505124	PI + Bleomycin + SB505124 + Navitoclax
	0	33.0	75.3%	86.6%
5			PI + PLX4720 + Navitoclax	PI + PLX4720 + Navitoclax + Roscovitine
	0	33.0	75.3%	84.5%
6			PI + Afatinib + Navitoclax	PI + Afatinib + Navitoclax + MLN4924
	0	33.0	72.2%	82.5%
7			PI + PD.173074 + MLN4924	PI + PD.173074 + MLN4924 + KIN001.055
	0	33.0	71.1%	82.5%
8			PI + SN.38 + SB505124	PI + SN.38 + SB505124 + ATRA
	0	33.0	73.2%	85.6%
9			PI + Bicalutamide + Navitoclax	PI + Bicalutamide + Navitoclax + EHT1864
	0	33.0	72.2%	82.5%
10			PI + MLN4924 + PIK.93	PI + MLN4924 + PIK.93 + SB505124
	0	33.0	74.2%	84.5%
11			PI + UNC0638 + 17.AAG	PI + UNC0638 + 17.AAG + EHT1864
	0	33.0	72.2%	82.5%
12			PI + YM201636 + Temozolomide	PI + YM201636 + Temozolomide + AZD8055
	0	33.0	72.2%	82.5%
13			PI + Methotrexate + JW.7.24.1	PI + Methotrexate + JW.7.24.1 + AMG.706
	0	33.0	73.2%	84.5%
14			PI + KU.55933 + GSK269962A	PI + KU.55933 + GSK269962A + KIN001.055
	0	33.0	72.2%	83.5%
15			PI + NU.7441 + JQ1	PI + NU.7441 + JQ1 + EHT1864
	0	33.0	72.2%	82.5%
16			PI + AZD6482 + UNC0638	PI + AZD6482 + UNC0638 + MLN4924
	0	33.0	74.2%	84.5%
17			PI + CCT018159 + CP466722	PI + CCT018159 + CP466722 + JQ1
	0	33.0	72.2%	82.5%
18			PI + JQ1 + Doxorubicin	PI + JQ1 + Doxorubicin + 17.AAG
	0	33.0	74.2%	84.5%
19			PI + UNC0638 + AS605240	PI + UNC0638 + AS605240 + Roscovitine
	0	33.0	74.2%	83.5%
20			PI + YK4.279 + TL.2.105	PI + YK4.279 + TL.2.105 + Temsirolimus
	0	33.0	73.2%	82.5%
21			PI + AICAR + SN.38	PI + AICAR + SN.38 + SB505124
	0	33.0	71.1%	83.5%
22			PI + Docetaxel + Bleomycin	PI + Docetaxel + Bleomycin + Roscovitine
	0	33.0	72.2%	83.5%
23			PI + PD.0332991 + Gefitinib	PI + PD.0332991 + Gefitinib + Bicalutamide.1
	0	33.0	71.1%	80.4%
24			PI + AG.014699 + Trametinib	PI + AG.014699 + Trametinib + Roscovitine
	0	33.0	71.1%	81.4%
25			PI + GSK269962A + Navitoclax	PI + GSK269962A + Navitoclax + Cetuximab
	0	33.0	71.1%	81.4%
26			PI + piperlongumine + CP466722	PI + piperlongumine + CP466722 + MLN4924
	0	33.0	72.2%	80.4%
27			PI + Trametinib + CP466722	PI + Trametinib + CP466722 + SB505124
	0	33.0	72.2%	82.5%
28			PI + KIN001.055 + Temozolomide	PI + KIN001.055 + Temozolomide + Temsirolimus
	0	33.0	73.2%	82.5%

Percent coverage (cell lines predicted to be killed by each combination treatment regimen) of the cancer lines included in the prediction model is also provided.

### Top secDrugs induce loss of viability in HMCLs as single-agent treatment

**Fig. 1:**
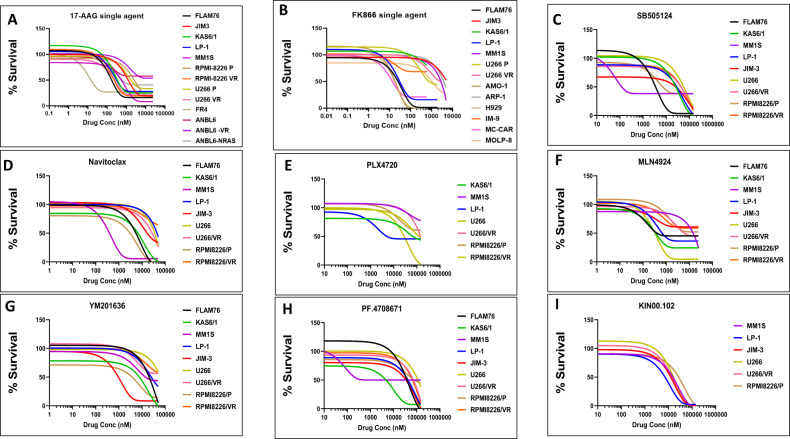
secDrugs decrease in vitro cell viability in multiple myeloma. Single-agent dose-response plots for the secDrugs in HMCLs. **A** 17-AAG; **B** FK866; **C** SB505124; **D** Navitoclax; **E** PLX4720; **F** MLN4924; **G** YM201636; **H** PF.4708671; **I** KIN001.002.

First, we used our panel of HMCLs as in vitro validation screens to evaluate the top predicted secDrugs, including 17-AAG, PF.4708671, SB505124, Navitoclax, PLX4720, MLN4924, YM201636, FK866, KIN001.002. As shown in Fig. 1, the predicted secDrugs showed high single-agent in vitro cytotoxicity in our myeloma cell line panel, including innate and acquired PI-resistant and IMiD-resistant myeloma cell lines compared to untreated control at increasing concentrations of secondary drugs.

### Single-cell transcriptomics (scRNA-seq)-based drug screening predicted 17-AAG + FK866 as potentially effective against myeloma

**Fig. 2:**
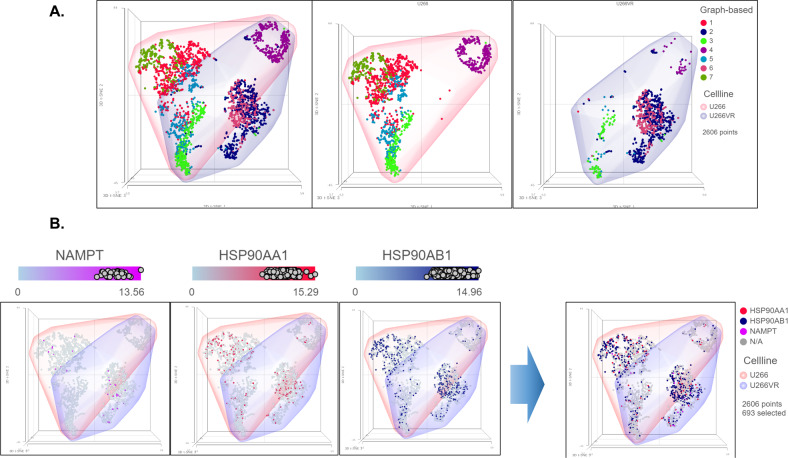
Representative plots showing single-cell RNAseq analysis results in sensitive and acquired-resistant myeloma cell line pairs. **A** Comparison of the t-SNE/Graph-based clusters between U266P vs. U266VR cell lines (U266P—parental/sensitive, U266VR—acquired-resistant). **B** Figure showing single-cells with an enriched expression of the target genes of 17AAG (HSP90AA1, HSP90AB1) and FK866 (NAMPT).

Next, we used single-cell RNA sequencing (scRNAseq) as a novel biomarker-based drug screen to identify single-cell sub-clones (represented by t-SNE clusters) that harbor secDrug target genes in the untreated/baseline HMCLs representing sensitive or myeloma tumors. Our scRNA-seq data in (representative t-SNE clusters shown in Fig. 2) demonstrated that the majority of the single-cell clusters in drug-sensitive and drug-resistant myeloma have high expression of 17-AAG target genes HSP90AA1, HSP90AB1, and the FK866 target gene NAMPT indicating that 17-AAG and FK866 combination may be effective against these subpopulation clusters. The 17-AAG target gene list was derived from the Harvard Medical School (HMS)’s NIH Library of integrated network-based cellular signatures perturbagen database, a publicly available database devoted to understanding how human cells respond to perturbation by drugs, the environment, and mutation [23, 24].

### 17-AAG shows synergy with PIs, IMiDs, and FK866

**Fig. 3:**
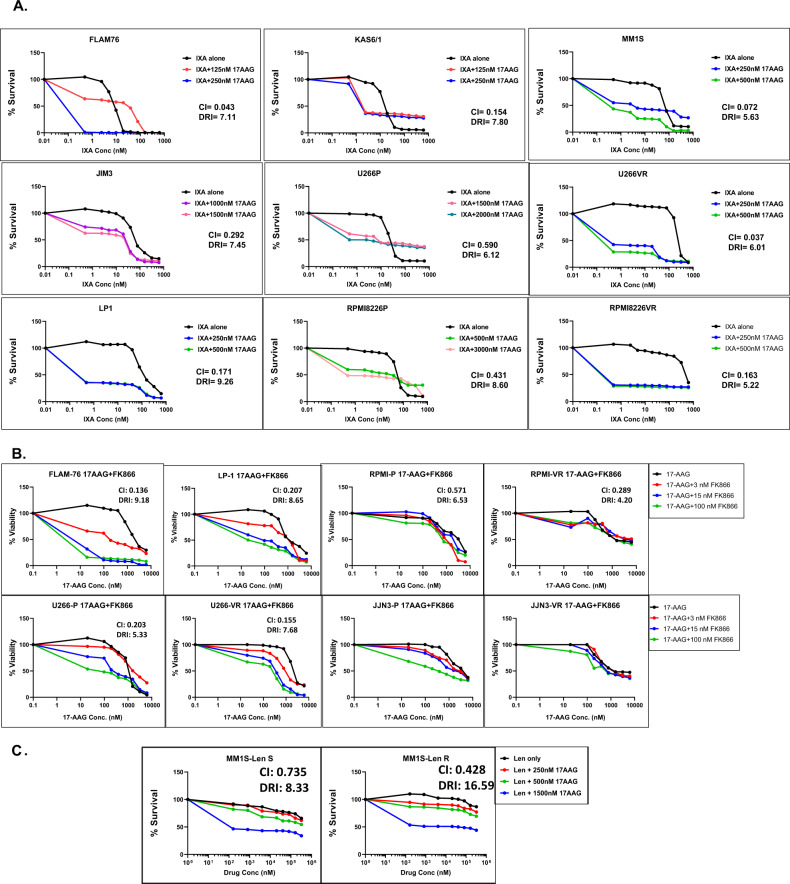
The secDrug 17AAG (Hsp90 inhibitor) synergizes with A. PIs (17AAG + IXA), B. FK866 (17AAG + FK866), and C. IMiDs (17AAG + Lenalidomide). In vitro dose–response plots for secDrug combination treatment in HMCLs representing innate sensitivity, innate resistance, Parental/sensitive, and clonally derived PI/IMiD acquired resistance. Cell viability was assessed by CellTiter-Glo assay (48 h). CI (combination index) and DRI (dose reduction index) values were calculated using Chou–Talalay’s CI theorem. (CI > 1—antagonism; CI = 1—additive; CI < 1—synergism) (VR-velcade/bortezomib/PI-resistant cell lines, LenR- Lenalidomide/IMiD-resistant cell line).

We used a sub-panel of HMCLs representing PI-sensitive (FLAM76, KAS6/1, MM1S), innate resistance (JIM-3, LP-1; representing refractory disease), and acquired PI/IMiD resistant clonal pairs (U266P/VR, RPMI8226P/VR, JJN-3P/VR, and MM1SP/LenR; representing relapse MM) to evaluate the effect of the predicted secDrug-based combination regimen, 17-AAG + FK866 either as a combination of these two secDrugs or using PIs or IMiDs as the backbone. Cell survival curves representing 17-AAG + Ixazomib, 17-AAG + FK866, and 17-AAG + Lenalidomide combination are shown in Fig. 3A–C. We found that 17-AAG not only showed synergy with PIs and IMiDs, the combination of 17-AAG and FK866 also showed significant synergy, as depicted by the dose-response curves and CI values representing combination treatments. CI values were consistently less than 1, which indicates synergy [25]. In addition, FK866 also showed synergy with Ixazomib (Ixa + FK866 survival curves are shown in Fig. S1A). Cell survival curves representing other top secDrugs + Ixazomib combination in innate sensitive, innate resistant, and acquired resistant HMCLs are shown in Fig. S1B–F. These secDrugs also showed strikingly high synergy with Ixazomib, as depicted by the CI values. Further, based on dose reduction index (DRI) values, the IC_50_ of Ixazomib in myeloma cell lines was predicted to be significantly reduced in the presence of these secDrugs.

Figure S2 shows the relative decrease of the predicted effective IC_50_ (nM concentration) of Ixazomib when used in combination with 17-AAG. The DRI values, calculated using CI theorem, demonstrated that 17-AAG improved the therapeutic index of PI and IMiD administration to the cells and decreased the amount of PI/IMiD required to achieve effective responses [25]. This points towards the possibility of reducing the dose and thereby toxicity of PIs when administered as 17AAG + PI combination. Drug-induced apoptosis was confirmed in HMCLs using Caspase 3/7 activity assays (data not shown).

### CyTOF analysis revealed 17-AAG-induced cell death of PMCs and key changes in myeloma-specific proteomic markers

**Fig. 4:**
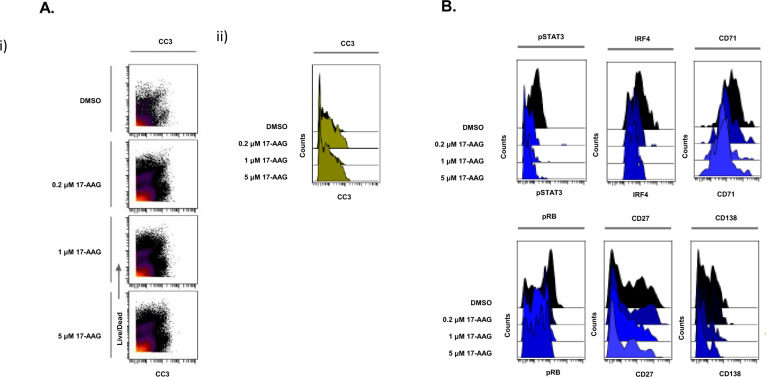
Representative figures showing CyTOF analysis results in patient primary multiple myeloma cells. CyTOF analysis was performed on Live cells (*n* = 6). **A** 17-AAG induces elevated cleaved caspase 3 levels. Samples were treated with 17-AAG (2, 5, and 10 μM) or DMSO and Gated on LIVE cells. (i) The FlowSOM meta-cluster results were condensed into cc3 positive and negative cell subsets based on cc3 expression UMAPs and plotted over CLF dose. (ii) cc3 induction is also shown in the violin plots. **B** Downregulation of genes associated with myeloma cell survival. Representative violin plots of CyTOF analysis in patient primary myeloma cells showing expression of myeloma markers following 17-AAG treatment, including IRF4, pSTAT3, IZKF3, CD138, CD71, pRB, and CD27.

High-dimensional mass cytometry or CyTOF analysis is a deep immunophenotyping method that combines flow cytometry and elemental mass spectrometry [26]. We performed CyTOF analysis on PMCs obtained from myeloma patients (*n* = 6) to assess 17-AAG-induced cell death through apoptosis as well as to evaluate changes in phenotypic and functional markers in MM cells at the single-cell/subclonal proteomics levels. As shown in Fig. 4A, CyTOF analysis following exposure to 17-AAG treatment revealed a distinct cluster of cells defined by elevated cleaved caspase levels in the primary samples, indicating treatment-induced cell death by apoptosis in the cells exposed to 17-AAG.

Myeloma cells are addicted to several proteins. Figure 4B shows results from our CyTOF-based differential expression (pre vs. post-17-AAG treatment) analysis, revealing shifts in IRF4 and pSTAT3 as well as myeloma cell survival markers like CD138 and phosphoproteins like pRB.

### Western blotting analysis

Treatment-induced protein expression of the phenotypic/functional markers of myeloma (p65/NFκB, IRF4, and cMyc) and markers of apoptosis (including Cleaved Caspase-3, Cleaved Caspase-9, etc.) was confirmed using immunoblotting analysis in HMCLs. Figure S3A shows representative pre- vs. post- 17-AAG treatment immunoblotting results on these myeloma cell survival and apoptotic pathways. Densitometry analysis results are provided in Fig. S3B. Our results show a substantial decrease in IRF4, p65, and cMyc following 17-AAG treatment and a concurrent increase in Cleaved Caspase-3, Cleaved Caspase-9 protein expression, which was also confirmed at the mRNA level using pre- vs. post-17AAG-treatment differential gene expression (RNAseq) analysis, along with several other myeloma protein/survival markers like STAT1, RELB, NFKBIA, NFKB2, and IKZF3.

### 17-AAG induces apoptosis via a mitochondrial-mediated pathway in myeloma

To investigate if 17-AAG imparts its cytotoxic effects in myeloma by generating reactive oxygen species (ROS), particularly superoxides and hydrogen peroxide (H_2_O_2_), cellular superoxide anions were measured by using the fluorescent dye DHE (Abcam). MMP was assessed using JC-1 (Abcam). JC-1 is a cationic carbocyanine dye that accumulates in mitochondria. The dye exists as a monomer (green fluorescence) at low concentrations and changes color from green to red in energized mitochondria.

**Fig. 5:**
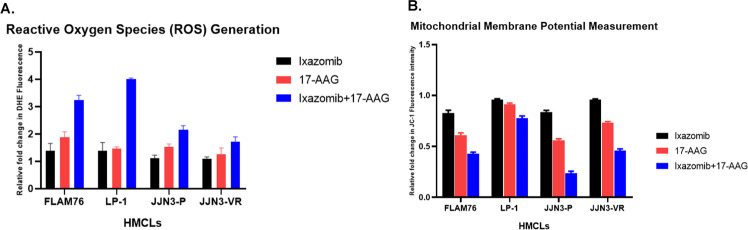
17-AAG induces super-oxide levels, intracellular ROS generation, and mitochondrial membrane potential (MMP) in myeloma cell lines. **A** Super-oxide. Cellular superoxide anions were measured by using the fluorescent dye DHE (Abcam), and red fluorescence was detected by Synergy Neo2 multi-plate reader. **B** Mitochondrial membrane potential (MMP) was assessed using JC-1 (Abcam), a cationic carbocyanine dye that accumulates in mitochondria. The dye exists as a monomer (green fluorescence) at low mitochondrial membrane potential and changes color from green to red in energized mitochondria. Cells were incubated with 5 μM JC-1 dye for 15 min in the dark at 37 °C, washed twice in PBS, and then analyzed for red and green fluorescence by Synergy Neo2 multi-plate reader.

We observed induction of cellular superoxide anions (Fig. 5A) and intracellular ROS production (Fig. 5B) that causes mitochondrial membrane depolarization following 17-AAG treatment in myeloma cells representing sensitive and resistant disease.

### 17-AAG induced cell death was comparable with Hsp90 knockdown

Next, we compared the effect of Ixa and Ixa+17-AAG combination therapy between wild-type and CRISPR-mediated HSP90AA1 gene knockdown cell lines. Dose–response curves in Fig. S4 show that the in vitro cytotoxicity in RPMI8226 cell lines was comparable following HSP90 inhibition, either through 17-AAG treatment or CRISPR-mediated HSP90 knockdown. This points toward an on-target effect of 17-AAG treatment leading to the anti-MM efficacy.

### NRas mutant HMCL showed high sensitivity to 17-AAG treatment

Finally, the myeloma cell lines ANBL6P, ANBL6VR, and ANBL6 NRas mutant were treated with single-agent 17-AAG, Ixa, and 17-AAG + Ixa combination. We have described earlier that these activating mutations of the ras oncogenes in ANBL6 (ANBL6 NRas) may lead to growth factor independence and suppression of apoptosis [18]. Notably, our ANBL6 NRas mutant cell line showed 20-times greater 17-AAG sensitivity (lower IC_50_) compared to the ANBL6P or VR cell lines (Fig. S5).

### Gene-expression profiling analysis results

First, we compared the baseline (untreated) bulk mRNA sequencing analysis profiles of the HMCLs representing extraordinary responses (top-most sensitive vs. top-most resistant) to 17-AAG. At *p* < 0.05, next-generation mRNA sequencing analysis showed 421 genes were DE between the 17-AAG-sensitive and the 17-AAG-resistant groups (fold-difference≠1). Among these, 360 genes had a fold change difference of >2 or <−2 between sensitive and resistant groups. Table S2 shows the top 50 genes (top 25 upregulated + top 25 downregulated) that were most DE as signatures of 17-AAG sensitivity in myeloma. IPA analysis revealed B Cell Receptor Signaling (*p* = 1.90E−03), RhoGDI Signaling (*p* = 1.22E−03), and IL-10 Signaling (*p* = 1.43E−03) as the top canonical pathways associated with 17-AAG sensitivity in myeloma based on the genes that were DE.

**Fig. 6:**
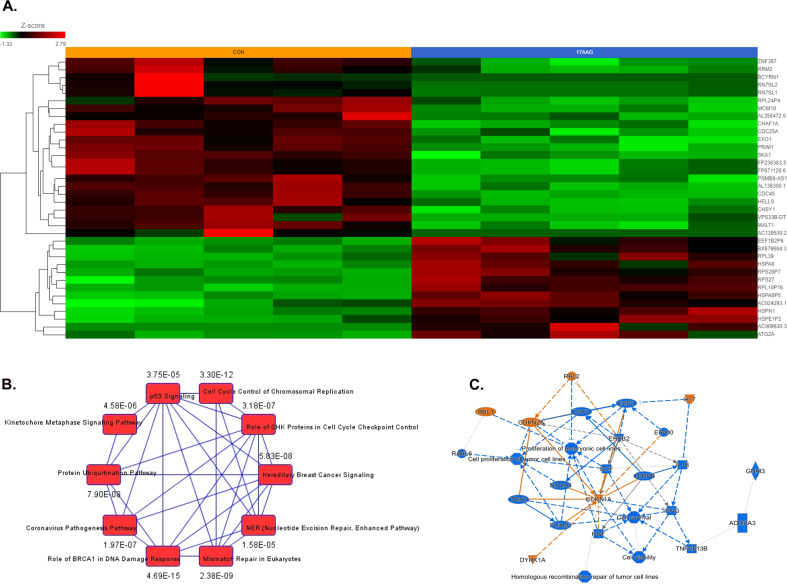
Differential gene expression analysis of 17-AAG single-agent treatment. **A** Heatmaps generated using unsupervised hierarchical clustering (HC) analysis showing top differentially expressed genes (bulk RNAseq data) that showed significant de-regulation 24 h following Single-agent 17-AAG exposure. IPA analysis results show **B** canonical pathways and **C** graphical summary. Columns represent cell lines, and rows represent genes. Prior to Hierarchical clustering, gene expression values were *z*-score normalized.

Differential gene expression analysis of kinetic (treatment-induced) changes between baseline (untreated) vs. single-agent 17-AAG (0.5 μM) treatment (24 h) in HMCLs representing sensitive + intermediate + resistant myeloma showed a total of 1449 genes were DE in response to 17-AAG with *p*-value less than 0.05 (|fold-change | >1). Among these, 865 genes had a |fold-change | >2. Figure 6A shows a heat map of the top 36 DEGs (|fold-change | >1; false-discovery rate (FDR < 0.05)). When single-agent 17-AAG-induced kinetic changes were considered separately for each HMCL (RPMI8226, FLAM76, JIM3, U266, and LP1), 422 genes were found common between all the Treated vs. Untreated signatures at |fold-change | >2 (*p* < 0.05), as shown in the Venn diagram (Fig. S6). IPA analysis (Fig. 6B) based on the DEG signatures of 17-AAG single-agent treatment revealed cell cycle control of chromosomal replication (*z*-score −4.243; *p*-value 3.30E−12), EIF2 signaling (2.496; *p* = 1.12e−04), aryl hydrocarbon receptor signaling (*z*-score −3.464; *p*-value 1.96E−03), and protein ubiquitination pathway (PUP; *p* = 7.90e−08) as top canonical pathways. Downregulation of CEBPB (*z*-score −6.670; *p*-value of overlap 5.28e−19), ERBB2 (*z*-score −5.358; *p*-value 2.57e−08), CSF2 (*z*-score −4.750; *p*-value 1.24e−05) and CCND1 (*z*-score −3.707; *p*-value 2.40e−07) and upregulation of the microRNAs let-7 (*z*-score 5.501; *p*-value 2.01e−09) were predicted as the top upstream regulator based on significantly DEGs (Fig. 6C). Interestingly, IPA analysis also showed that gene signatures of 17-AAG treatment were positively correlated with that of bortezomib (*z*-score 2.048; *p*-value 1.68e−05) and lenalidomide (*z*-score 2.774; *p*-value 2.80e−02), indicating a possible basis for 17-AAG + PI and 17-AAG + IMiD synergy.

**Fig. 7:**
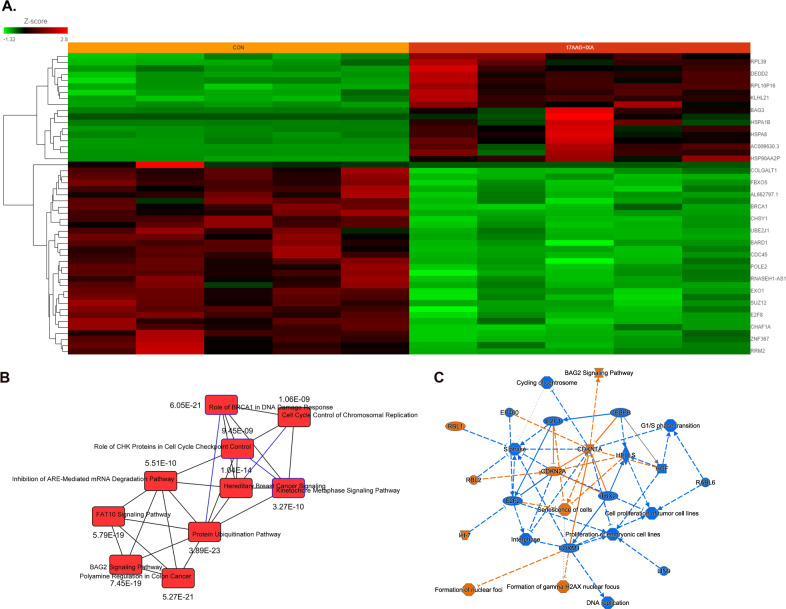
Differential gene expression analysis of 17-AAG + PI combination treatment. **A** Heatmaps generated using unsupervised hierarchical clustering (HC) analysis showing top differentially expressed genes (bulk RNAseq data) that showed significant de-regulation, 24 h following 17-AAG + Ixazomib combination treatment. IPA analysis results show **B** top 10 canonical pathways and **C** graphical summary. Columns represent cell lines, and rows represent genes. Prior to Hierarchical clustering, gene expression values were *z*-score normalized.

A total of 3974 genes changed significantly between untreated vs. 17-AAG + Ixa combination-treated samples (*p* < 0.05; fold-difference≠1). Among these, 853 genes showed |fold-change | >2 with a FDR < 0.05. Figure 7A depicts a heatmap of the top 50 genes associated with 17-AAG + Ixa combination treatment. IPA analysis based on DEGs significantly associated with 17AAG + Ixa treatment revealed PUP (*p* = 3.89E−23) as the top canonical pathway (Fig. 7B). Upstream regulator prediction analysis revealed inhibition of the transcriptional regulators CEBPB (*z*-score −8.871; *p*-value of overlap 1.38e−22), MYC (*z*-score −6.732; *p*-value of overlap 3.83e−18), as well as VEGF (*z*-score −6.805; *p*-value 9.76e−07), HGF (*z*-score −7.139; *p*-value 2.08e−10), and CSF2 (*z*-score −6.770; *p*-value 4.16e−07) following 17-AAG + PI combination treatment (Fig. 7C).

The Venn diagram in Fig. S7A shows 50 genes were common between the three comparisons (17-AAG vs. Control, 17-AAG + Ixa vs. Control, and Ixa vs. control). Further, Fig. S7B also shows IPA-predicted canonical pathways that these 50 common genes (*p* < 0.05) represent.

Finally, IPA predicted upregulation of the microRNA let-7 (*z*-score 7.180; *p*-value 5.02e−12) as the top upstream regulator based on significantly DEGs (Fig. S8)

### Creation of secDrug software package

Finally, we developed an R software package based on our secDrug pipeline for predicting novel secondary therapies in chemotherapy-resistant cancers. secDrug takes a query of any cancer type and any test/primary/standard-of-care drug and outputs a list of the top secondary drug combinations with a confidence score and biological pathway visualization. Thus, secDrug has potential application in clinical decision-making for discovering resistance-reversing cancer chemotherapy regimens. R codes for the package are available at https://github.com/Ujjal-Mukherjee/secDrug/tree/main/CombinationDrugMyeloma, and the datasets are available at GitHub repository.

## Discussion

Drug resistance is a major obstacle in achieving a complete and sustained therapeutic effect in cancer chemotherapy [2, 11, 14, 27, 28]. Chemo-resistance may also lead to over-dosing and unwanted exposure to ineffective anti-tumor agents, thereby increasing the risk of negative side effects and the cost of drug development [29, 30].

In this study, we demonstrate the creation of a novel pipeline for drug development/drug repurposing that integrates *in silico* computational prediction, single-cell multi-omics (single-cell transcriptomics/scRNAseq and single-cell proteomics/CyTOF analysis) with in vitro and ex vivo validation, including the use of whole-genome transcriptomics (RNAseq) and genome editing technologies to identify and functionally validate secondary treatment regimens to circumvent drug resistance in myeloma. Notably, we applied the pipeline to predict several drugs as potential candidates for anti-MM secDrugs for combining with PIs. These (“top secDrugs”) include, HSP90 inhibitor/17-AAG, Nicotinamide phosphoribosyltransferase or Nampt-inhibitor/FK866, Survivin-inhibitor/YM155, PIKfyve-inhibitor/YM201636, Raf-inhibitor/PLX-4720, Bcl2-inhibitor/Navitoclax, AKT inhibitor/KIN00102, transforming growth factor-β type I receptor, ALK4, ALK7-inhibitor/SB505124, HDAC-inhibitors (Panobinostat, SAHA), S6K1-specific inhibitor/PF-4708671, and the neddylation-inhibitor/MLN4924.

Further, we performed extensively in vitro, ex vivo, and functional validation in research models of refractory and resistant myeloma to validate 17-AAG + FK866, 17AAG + PI, and 17-AAG + IMID as combination treatment candidates that also served as a proof-of-principle for our secDrug pipeline. Overall, our validation results corroborated with our in silico prediction of secondary drugs based on secDrug analysis.

17-AAG/Tanespimycin has previously been shown to work against myeloma, in vitro, in vivo (animal studies) as well in clinical studies [31–34]. However, to our knowledge, this is the first study that specifically evaluates the use of 17-AAG combination therapy in relapsed and refractory myeloma models. Further, in our study, the ANBL6 Ras mutant cell line showed 20-times lower 17-AAG cytotoxicity compared to the ANBL6P/VR cell lines. An earlier study in metastatic malignant melanoma has shown that a patient harboring NRAS-activating mutation exhibited disease stabilization for 49months following administration of pharmacologically active doses of 17-AAG [35]. Mutations of NRAS have earlier been shown to be significantly associated with lower single-agent PI-sensitivity and shorter time to progression in bortezomib-treated myeloma patients [36]. Thus, our study points towards a unique niche (NRas-mutant myeloma) where 17-AAG could be highly effective as a single agent as well as in combination with PIs and FK866, in addition to relapse and refractory myelomas. Moreover, we evaluated the molecular pathways involved in response to the top secondary drugs, which provided additional insights into the mechanism of action of 17-AAG as a secDrug.

Myeloma tumor cells have elevated intracellular NAD+ levels that support the high rate of energy metabolism for uncontrolled proliferation, tumor cell growth, and survival [37]. FK866 is a chemical inhibitor of Nampt (Nicotinamide phosphoribosyltransferase), a key enzyme in NAD+ metabolism [38]. Consequently, FK866 has been shown to reduce myeloma tumor growth in PI-sensitive and PI-resistant myeloma through activation of autophagy and cell death in myeloma cells [39]. In this study, we showed that FK866 not only overcomes PI-resistance when used as a single-agent or as an Ixa combination, combining 17-AAG + FK866 is highly synergistic against our validation models of relapsed/refractory myeloma.

Our study introduces several novels secDrugs as potential synergistic partners of PIs that have never been studied as potent single-agent or combination therapy options in myeloma model systems, including KIN001-102 (A6730; Akt1/2 kinase inhibitor) and SB505124 (inhibitor of transforming growth factor-β type I receptor or ALK4, ALK7 that activates the SMAD2/3 pathway). These may serve as novel candidates for further studies on the pre-clinical and clinical validation in xenograft or mouse models of myeloma.

Although some of the other predicted secDrugs have earlier been shown to be effective against myeloma, very few studies have explored their efficacy as drug combinations with PIs/IMiDs in models of refractory/resistant myeloma. For example, PF-4708671 is a P70S6K1 isoform-specific inhibitor that has recently been shown to induce statistically significant apoptosis in HMCLs and PMCs in combination with several standard-of-care therapies [40]. NEDD8-activating enzyme/neddylation-inhibitor/MLN4924 has once earlier been shown effective against a subset of cell lines represented by cell surface expression of TNFR1 [41]. PLX4720 (a small-molecule, ATP-competitive inhibitor of Mutant BRAF kinase) was earlier shown to have a partial single-agent response in patients harboring subclonal *BRAF* mutations [42, 43]. Our in silico predictions and single-agent cytotoxicity data thus builds a strong case to test these drugs as secDrug combination regimens in a wider panel of HMCLs representing refractory and clonally derived acquired resistant cell lines. Among the other secDrugs, Navitoclax is a high-affinity small-molecule BH3 mimetic that inhibits Bcl2 and Bcl-xl. Navitoclax has been shown to inhibit cell proliferation in myeloma leading to induction of apoptosis [44, 45]. YM201636 is an inhibitor of PIKfyve, a mammalian protein involved in the regulation of crucial cellular functions, including nuclear signaling and autophagy. Few recent studies demonstrated the therapeutic efficacy of PIKfyve inhibitors in myeloma cell lines [46, 47].

Overall, we present here a unique pipeline that introduces not only novel secDrugs but also provides additional niches for secondary drugs that are already under preclinical or clinical investigation in relapsed/refractory myeloma.

Our findings provide a strong case for combining the top predicted secDrugs with PIs and IMiDs to overcome resistance and thereby improve patient outcomes. This potentially introduces many more drugs as new and more effective therapeutic options for the management of resistant myeloma with a high probability of clinical success that promises to improve the quality of treatment, maximize drug efficacy, minimize toxicities and adverse drug reactions from over-dosing and decrease the rate of mortality in myeloma patients. A logical extension of this pipeline would be the development of model systems where a combination of more than two secDrugs can be effectively tested.

The integration of in silico modeling-based pipeline with single-cell technologies (scRNAseq and CyTOF analysis) introduces an innovative, evidence-based application in clinical decision-making that will minimize the number of test drugs required for discovering successful combination chemotherapy regimens against drug-resistant cancers.

## Supplementary information

Supplementary Methods

Supplementary Tables

Supplementary Figures

## Data Availability

All data used in the analysis are available on reasonable request from the corresponding author.

## Code availability

All code used in the analysis is available on reasonable request from the corresponding author.

## Materials availability

All materials used in the analysis are available on reasonable request from the corresponding author.
