# Supplementary material for: secDrug: a pipeline to discover novel drug combinations to kill drug-resistant multiple myeloma cells using a greedy set cover algorithm and single-cell multi-omics
Source: Blood Cancer J. 2022 Mar 9;12(3):39. doi: 10.1038/s41408-022-00636-2 (PMC8907243; doi:10.1038/s41408-022-00636-2)
Supplement: Supplementary file 1 — Supplementary Methods [file 41408_2022_636_MOESM1_ESM.docx]

**SUPPLEMENTARY METHODS**

***In silico* prediction** **of secondary drugs (secDrug pipeline)**

We utilized a data-driven greedy algorithm-based set-covering computational optimization method followed by a regularization technique to seek all secondary drugs (secDrugs) that could kill the maximum number of cell lines of the test disease (B-Cell cancers) resistant to the test drug (the Proteasome inhibitor/PI drug Bortezomib/Bz/Velcade) in a sequential manner ordered by the number of cell lines killed. A greedy algorithm constructs a solution to an *optimization problem* piece by piece through a sequence of choices to find the overall, or globally, optimal solution. Towards this goal, we used the vast array of human cancer cell lines in the Genomics of Drug Sensitivity in Cancer (GDSC version GDSC1000) database. The Genomics of Drug Sensitivity in Cancer (GDSC1000) resource is the largest public collection of information on drug sensitivity in human cancer cells^1,2^. It provides a comprehensive database of drug-cell line combinations that incorporates drug-sensitivity data on 265 drugs covering a wide range of targets and processes involved in cancer biology on more than 1000 human tumor cell lines, representing a wide spectrum of human cancers, along with a wide array of genetic information (including gene expression analysis data)^1,2^. This provided us with a unique resource to investigate a wide variety of drugs as secondary choices in combination with PIs, which are: 1) approved and used in the clinic, or 2) drugs undergoing clinical development, or 3) drugs in clinical trials or 4) tool compounds in early phase development.

Briefly, let us assume that IC_50_ values of Bz in B-Cell lines (including HMCLs) are$S_{bi}:i\in\{1,\cdots,n\}$, where there are $n$ cell lines. Also, let us assume that there are $K$ other drugs and the IC_50_ values of the $n$ cell lines for the $K$ drugs are given by:

$R_{ki}:k\in\left\{ 1,\cdots,K \right\}, i\in\{1,\cdots,n\}$.

The first step was to classify the cell lines as sensitive or resistant to Bz. There are several ways in which this classification could be achieved. A simple and naïve approach would be to use a quantile of the empirical distribution of $R_{ki}$, and use a threshold criterion to achieve the classification. The advantage of this method is simplicity and interpretability. However, a primary disadvantage of this method is that it is ad-hoc, and the final result may be dependent on the subjective choice of the threshold, which did not use the pattern of the data. Therefore, we adopted an alternative method and performed the splitting using a threshold $\theta_{k}$ to minimize a loss function such as the squared loss as in the following equation,

$$\min_{\theta} \frac{\sum_{i=1}^{n^{\geq\theta}} {(R}_{ik}^{\geq\theta}-\bar{R}_{ik}^{\geq\theta})^{2}}{n^{\geq\theta}}+ \frac{\sum_{i=1}^{n^{<\theta}} {(R}_{ik}^{<\theta}-\bar{R}_{ik}^{<\theta})^{2}}{n^{<\theta}}.$$

In the loss function above, we minimized the sum of the squared loss (variance) of the data for the individual groups. This criterion is similar to a decision tree splitting criterion for continuous response variables. It is possible to use an overall variance reduction criterion for the entire sample; however, we use this criterion because it achieves a balanced variance reduction for both the sensitive and the resistant groups. The superscripts for each of the quantities indicate the selection condition of the quantity. This condition was similar to a decision clustering approach. The advantage of the squared loss minimization approach was that it used the data structure and was non-parametric, thereby not requiring parametric distributional assumptions. However, the primary disadvantage was that this method was sensitive to outliers and few large deviations in the data. Finally, we considered that the observed data $R_{ik}$ consisted of the latent variable, which indicated high or low response of cell lines to Bz. The observed data was a manifestation of the latent variable. We used a Gaussian mixture model estimation for computing the parametric distribution of the sensitive and resistant cell lines. However, an issue that needed accounting for was the data distribution. The raw data $R_{ik}$ was not normally distributed as estimated using the Wilk-Shapiro test. Therefore, we used the log-transformed response variable for the classification task. We denoted the latent variable of resistant and sensitive cell-line as $X= \{R, V\}$, where R denoted sensitive cell-line, and V denotes resistive cell line. Also, the log-transformed response variable was denoted as $Y_{ik}=log(R_{ik})$. We model the observed response as

$$Y_{ik}\sim\pi\mathbb{N}\left( Y_{ik} | \mu_{R}, \sigma_{R}^{2} \right)+\left( 1-\pi\right)\mathbb{N}\left( Y_{ik} | \mu_{S}, \sigma_{S}^{2} \right).$$

In the above model, $\mathbb{N}\left( Y_{ik} | \mu_{R}, \sigma_{R}^{2} \right)$ was a normal distribution representing the distribution of the $R$ class, and $\mathbb{N}\left( Y_{ik} | \mu_{S}, \sigma_{S}^{2} \right)$ was the distribution of the S class. The probability of an observation to be from the R class was $\pi$. Therefore, the probability that the observation was from the S class was $(1-\pi)$. The log-likelihood of the observed data was represented as

$$\mathcal{l}\left( \pi, \mu_{R}, \mu_{S}, \sigma_{R}, \sigma_{S} \right)= \sum_{i=1}^{n} \log\left( \pi\mathbb{N}\left( Y_{ik} | \mu_{R}, \sigma_{R}^{2} \right)+\left( 1-\pi\right)\mathbb{N}\left( Y_{ik} | \mu_{S}, \sigma_{S}^{2} \right) \right).$$

Since we did not observe the latent variable, we estimated the parameters from the above log-likelihood maximization problem by the Expectation-Maximization algorithm using the package *mixtools.* Based on the estimation of the mixture model, we classified the cell lines as sensitive and resistant. The classification based on the variance reduction criterion and the log-likelihood criterion matched fairly closely.

We indicated if a cell line was Bz-sensitive or resistant by the index $\mathcal{B}$, where $\mathcal{B}=1$ if a cell line was Bz-sensitive and zero otherwise. Also, we indicated whether a drug was in the combination (or not) by the variable: $z_{k}:k\in\{1,\cdots,K\}$, such that $z_{k}=1$ if the drug was in the optimal combination and zero otherwise.

*Therefore, the problem of creating the optimal combination for Bz resistant cell lines was that of a set cover problem, where the objective was to cover the set of Bz resistant cell lines by the minimal set of other drugs* ^3^*.*

Also, we denoted the set of sensitive cell lines to a drug $k$ that are not sensitive to Bz be denoted by: $\left[ \xi_{k} | \mathcal{B}_{k}=0 \right]=\{i:i\left( k \right)=1\}$.

The problem can be stated as:

$minimize \sum_{k} z_{k}$,

Subject to the conditions:

$$\left| \bigcup_{k} \left[ \xi_{k} | \mathcal{B}_{k}=0 \right]z_{k} \right|\geq\alpha\left| \bigcup_{k} \left[ \xi_{k} | \mathcal{B}_{k}=0 \right] \right|$$

$$\alpha\in\{0,1\}$$

Where $|\cdot|$denoted the cardinality of the set of cell lines that were already covered, and $\alpha$ was the fraction of cell lines that we intended to cover in a specific combination.

The set cover problem is NP-hard and does not have a closed-form polynomial-time solution^3,4^. We used a Greedy algorithm to solve the problem. The strategy of the greedy algorithm was to include that drug in the combination that covered the maximum number of cell lines that have not been covered by the combinations so far at any step. In the case of ties, we chose the drug with the lower average IC_50_ values. In our drug combinations, we have taken the covering proportion parameter $\alpha$ to be 0.95. Higher of lower values were chosen based on the requirement. Another way of choosing was using a gain criterion such that we chose that alpha beyond which additional gain by including new drugs was below a threshold value.

**References**

1 Garnett MJ, Edelman EJ, Heidorn SJ, Greenman CD, Dastur A, Lau KW *et al.* Systematic identification of genomic markers of drug sensitivity in cancer cells. *Nature* 2012; **483**: 570–575.

2 Yang W, Soares J, Greninger P, Edelman EJ, Lightfoot H, Forbes S *et al.* Genomics of Drug Sensitivity in Cancer (GDSC): a resource for therapeutic biomarker discovery in cancer cells. *Nucleic Acids Res* 2013; **41**: D955-61.

3 Caprara A, Fischetti M, Toth P. A Heuristic Method for the Set Covering Problem. *Oper Res* 1999; **47**: 730–743.

4 Moreno-Centeno E, Karp RM. The Implicit Hitting Set Approach to Solve Combinatorial Optimization Problems with an Application to Multigenome Alignment. *Oper Res* 2013; **61**: 453–468.
