# Supplementary material for: secDrug: a pipeline to discover novel drug combinations to kill drug-resistant multiple myeloma cells using a greedy set cover algorithm and single-cell multi-omics
Source: Blood Cancer J. 2022 Mar 9;12(3):39. doi: 10.1038/s41408-022-00636-2 (PMC8907243; doi:10.1038/s41408-022-00636-2)
Supplement: Supplementary file 2 — Supplementary Tables [file 41408_2022_636_MOESM2_ESM.docx]

**Supplementary Table S1: Immunophenotyping panel for CyTOF analysis**

**A. Cell surface targets**

| **SN.** | **Targets** | **Metal Tag** | **Source/Manufacturer** | **Catalog No.** |
| --- | --- | --- | --- | --- |
| 1. | CD45 | 89Y | Fluidigm | 3089003B |
| 2. | CD38 | 114Nd | Fluidigm | 3144014B |
| 3. | CD138 | 168Er | Fluidigm | 3168009B |
| 4. | CD3 | 141Pr | Fluidigm | 3141019B |
| 5. | CD56 | 149Sm | Fluidigm | 3149021B |
| 6. | CD19 | 169Tm | Fluidigm | 3169011B |
| 7. | CD81 | 145Nd | Fluidigm | 3145007B |
| 8. | CD20 | 147Sm | Fluidigm | 3147001B |
| 9. | CD34 | 148Nd | Fluidigm | 3148001B |
| 10. | CD274 | 159Tb | Fluidigm | 3159029B |
| 11. | CD27 | 167Er | Fluidigm | 3167006B |
| 12. | CD229 | 174Yb | Fluidigm | 3174017B |
| 13. | CD16 | 209Bi | Fluidigm | 3209002B |
| 14. | CD86 | 150Nd | Fluidigm | 3150020B |
| 15. | CD117* | 173Yb | BioLegend | 313223 |
| 16. | CD28* | 154Sm | BioLegend | 302937 |
| 17. | CD147* | 161Dy | BioLegend | 306206 |
| 18. | CD71* | 170Er | BioLegend | 334102 |

**B. Intracellular targets**

| **SN.** | **Targets** | **Metal Tag** | **Source/Manufacturer** | **Catalog No.** |
| --- | --- | --- | --- | --- |
| 1. | 1kBα | 164Dy | Fluidigm | 3164004A |
| 2. | pERK 1/2 [T202/Y204] | 171Yb | Fluidigm | 3171010A |
| 3. | pStat3 [Y705] | 158Gd | Fluidigm | 3158005A |
| 4. | IRF4 | 155Gd | Fluidigm | 3155014B |
| 5. | IKZF1 | 143Nd | Fluidigm | 3143024B |
| 6. | Ki-67 | 172Yb | Fluidigm | 3172024B |
| 7. | pS6 [S235/S236] | 175Lu | Fluidigm | 3175009A |
| 8. | MCL 1 | 163Dy | Fluidigm | 3163006A |
| 9. | Caspase 3/Cleaved | 142Nd | Fluidigm | 3142004A |
| 10. | pAkt [S473] | 152Sm | Fluidigm | 3152005A |
| 11. | p38 [T180/Y182] | 156Gd | Fluidigm | 3156002A |
| 12. | pRb [S807/811] | 166Er | Fluidigm | 3166011A |
| 13. | pCREB [S133] | 165Ho | Fluidigm | 3165009A |
| 14. | IKZF3 | 162Dy | Fluidigm | 3162032B |
| 15. | c-Myc | 176Yb | Fluidigm | 3176012B |
| 16. | Ig kappa/light chain | 160Gd | Fluidigm | 3160005B |
| 17. | Ig lambda/light chain | 151Eu | Fluidigm | 3151004B |
| 18. | BCL-2* | 153Eu | BioLegend | 658702 |
| 19. | Cyclin D1* | 146Nd | Santa Cruz Biotechnology | SC-8396 |

*In-house conjugated antibodies using the X8 polymer MaxPAR antibody conjugation kit (Fluidigm) as per manufacturer’s instructions.

**Supplementary Table S2: Top 50 (25 upregulated + 25 downregulated) differentially expressed genes between 17-AAG sensitive and resistant HMCLs**

| **Genes** | **Fold change (17AAG_Res vs. 17AAG_Sen)** | **P-value (17AAG_Res vs. 17AAG_Sen)** |
| --- | --- | --- |
| **PTPRCAP** | -183.73 | 2.56E-03 |
| **NAP1L3** | -102.49 | 9.77E-03 |
| **LTBR** | -97.47 | 1.46E-03 |
| **PITX1** | -88.67 | 1.81E-04 |
| **GABRB2** | -69.98 | 2.17E-02 |
| **RTKN2** | -67.63 | 4.26E-04 |
| **PCLO** | -63.18 | 3.40E-02 |
| **DYNC2H1** | -62.76 | 4.20E-02 |
| **CAPN5** | -62.71 | 1.65E-02 |
| **ARHGEF6** | -57.08 | 4.20E-03 |
| **PLEKHA5** | -54.43 | 1.46E-03 |
| **LGALS3BP** | -54.04 | 2.14E-02 |
| **KIAA1549** | -51.40 | 4.35E-04 |
| **CDC42BPA** | -40.97 | 1.11E-02 |
| **IGKC** | -38.91 | 3.60E-02 |
| **GBP2** | -36.97 | 1.68E-02 |
| **TRIM2** | -36.45 | 2.29E-03 |
| **HSPA12A** | -33.95 | 8.10E-03 |
| **EPB41L4A** | -31.70 | 4.25E-02 |
| **COL18A1** | -29.29 | 4.75E-02 |
| **WBP5** | -26.12 | 2.41E-02 |
| **CES3** | -24.97 | 1.21E-02 |
| **NRSN2** | -24.29 | 1.76E-02 |
| **DOK2** | -19.94 | 5.93E-03 |
| **SOX4** | -19.76 | 1.16E-02 |
| **HOMER3** | 15.61 | 1.38E-02 |
| **ASAP2** | 19.43 | 5.38E-03 |
| **PPDPF** | 19.67 | 2.41E-02 |
| **NLRP11** | 19.78 | 2.01E-02 |
| **ABCG2** | 21.88 | 1.12E-02 |
| **CDKN1C** | 22.68 | 3.89E-02 |
| **CLEC7A** | 25.90 | 1.62E-02 |
| **BIRC3** | 27.52 | 2.20E-02 |
| **HERC5** | 31.88 | 2.02E-02 |
| **AC005301.5** | 33.82 | 5.65E-03 |
| **CD74** | 40.50 | 4.40E-02 |
| **CD28** | 42.10 | 1.31E-03 |
| **RP11-844P9.2** | 48.85 | 1.69E-03 |
| **BLK** | 50.65 | 2.05E-02 |
| **ARL4C** | 55.17 | 3.78E-03 |
| **IFIT3** | 55.20 | 2.31E-02 |
| **SYNM** | 59.18 | 2.55E-02 |
| **HLA-DRA** | 61.95 | 2.26E-02 |
| **RGS1** | 76.85 | 3.87E-02 |
| **LAPTM4B** | 125.55 | 3.13E-02 |
| **FAM64A** | 156.03 | 1.54E-05 |
| **KCNK1** | 250.60 | 9.67E-03 |
| **RND3** | 324.32 | 3.78E-03 |
| **SCML1** | 788.94 | 3.19E-04 |
| **IGLC3** | 818.72 | 4.82E-03 |
