# Supplementary material for: secDrug: a pipeline to discover novel drug combinations to kill drug-resistant multiple myeloma cells using a greedy set cover algorithm and single-cell multi-omics
Source: Blood Cancer J. 2022 Mar 9;12(3):39. doi: 10.1038/s41408-022-00636-2 (PMC8907243; doi:10.1038/s41408-022-00636-2)
Supplement: Supplementary file 3 — Supplementary Figures [file 41408_2022_636_MOESM3_ESM.pptx]

## Slide 1
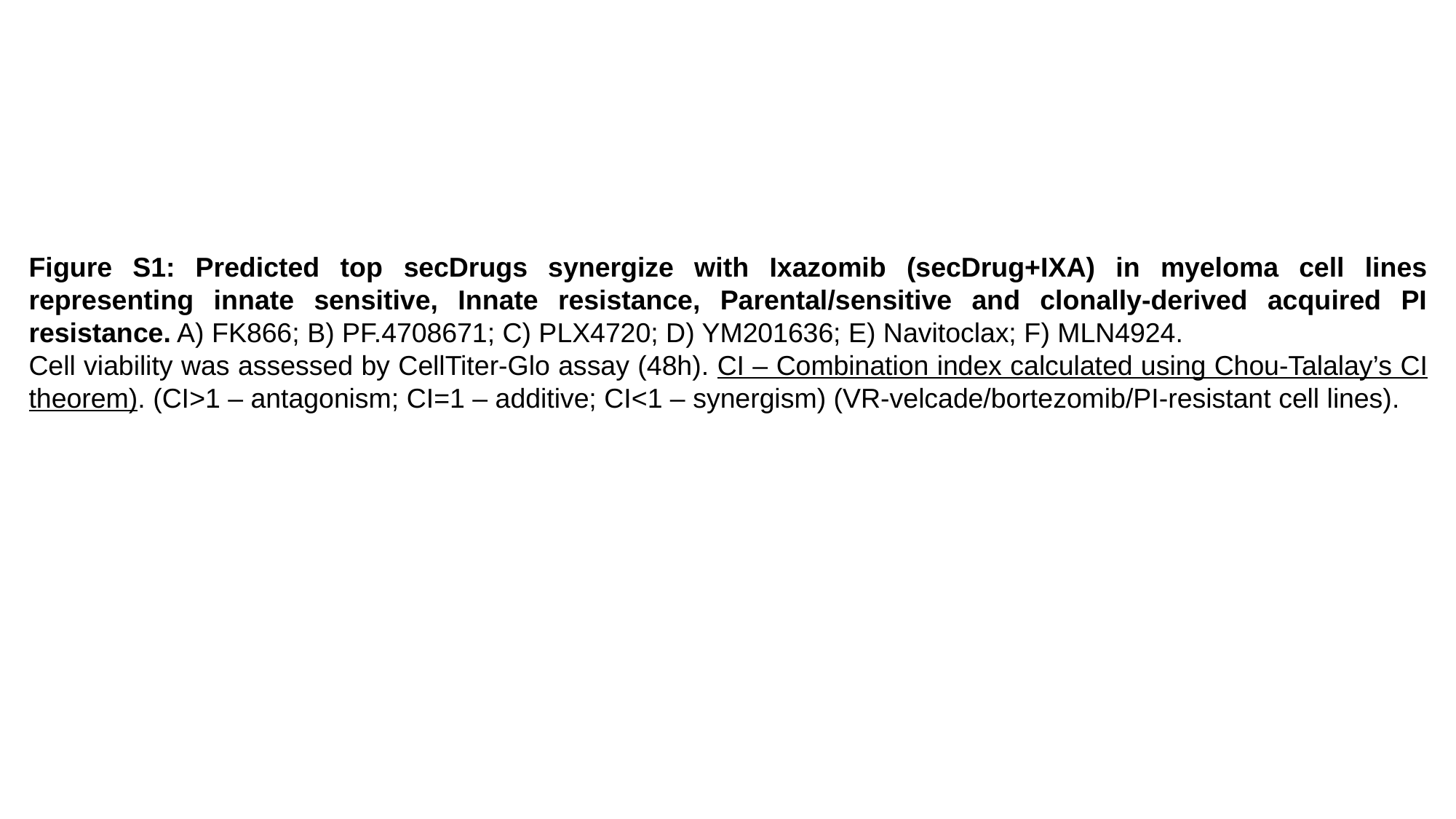

Figure S1: Predicted top secDrugs synergize with Ixazomib (secDrug+IXA) in myeloma cell lines representing innate sensitive, Innate resistance, Parental/sensitive and clonally-derived acquired PI resistance. A) FK866; B) PF.4708671; C) PLX4720; D) YM201636; E) Navitoclax; F) MLN4924.
Cell viability was assessed by CellTiter-Glo assay (48h). CI – Combination index calculated using Chou-Talalay’s CI theorem). (CI>1 – antagonism; CI=1 – additive; CI<1 – synergism) (VR-velcade/bortezomib/PI-resistant cell lines).

## Slide 2
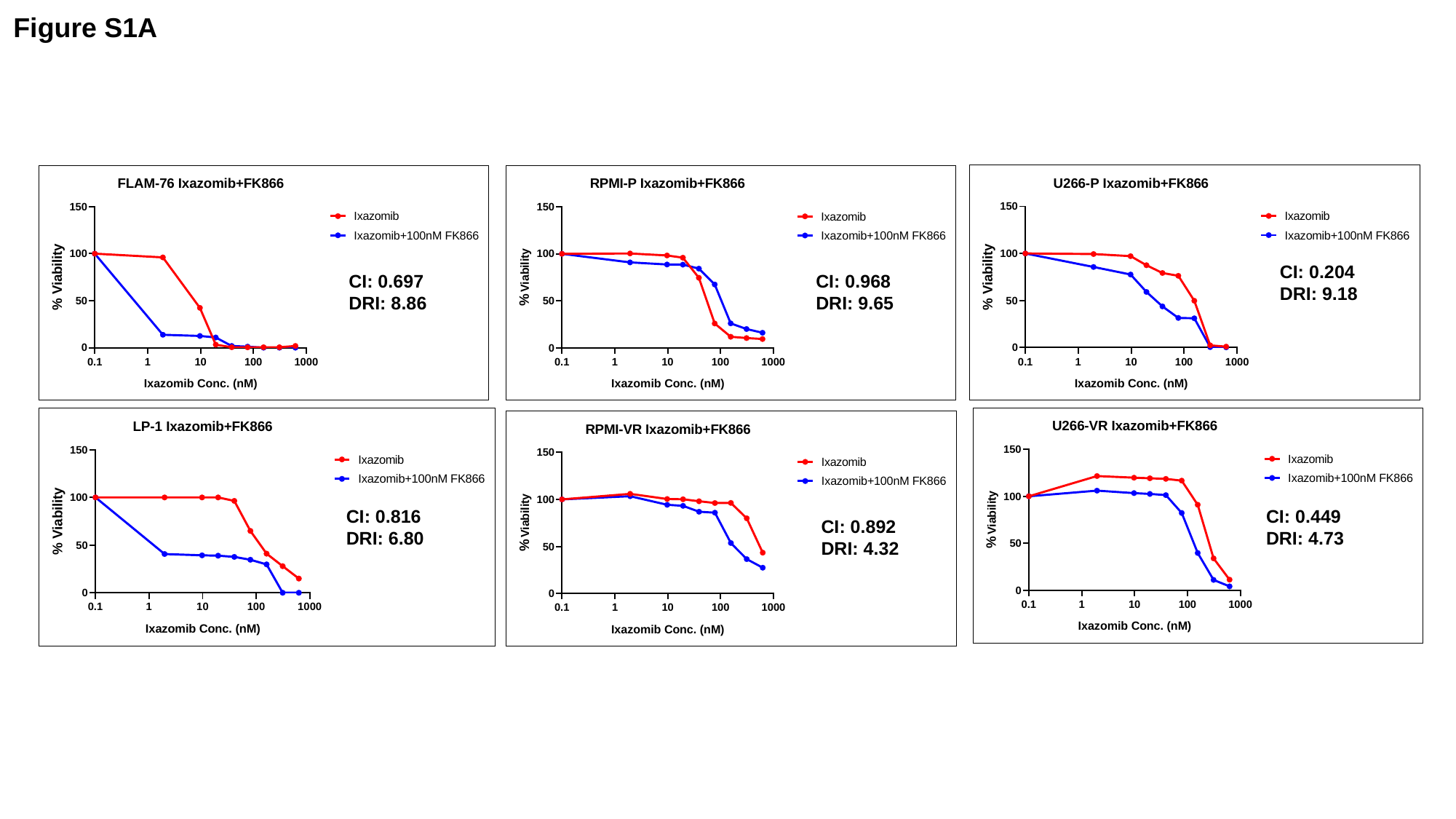

Figure S1A
CI: 0.204
DRI: 9.18
CI: 0.697
DRI: 8.86
CI: 0.968
DRI: 9.65
CI: 0.816
DRI: 6.80
CI: 0.449
DRI: 4.73
CI: 0.892
DRI: 4.32

## Slide 3
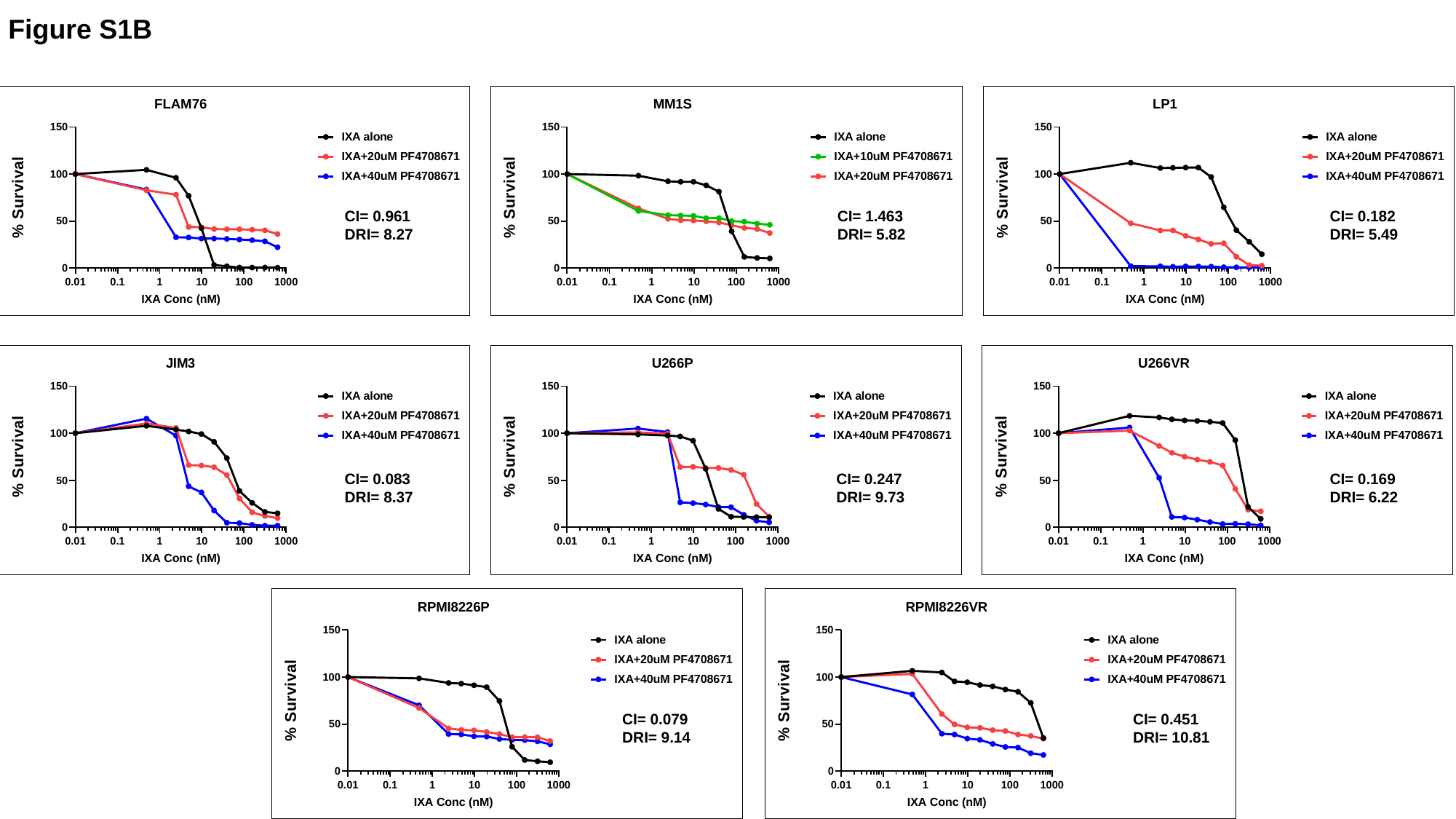

Figure S1B
CI= 0.961
DRI= 8.27
CI= 1.463
DRI= 5.82
CI= 0.182
DRI= 5.49
CI= 0.083
DRI= 8.37
CI= 0.247
DRI= 9.73
CI= 0.169
DRI= 6.22
CI= 0.079
DRI= 9.14
CI= 0.451
DRI= 10.81

## Slide 4
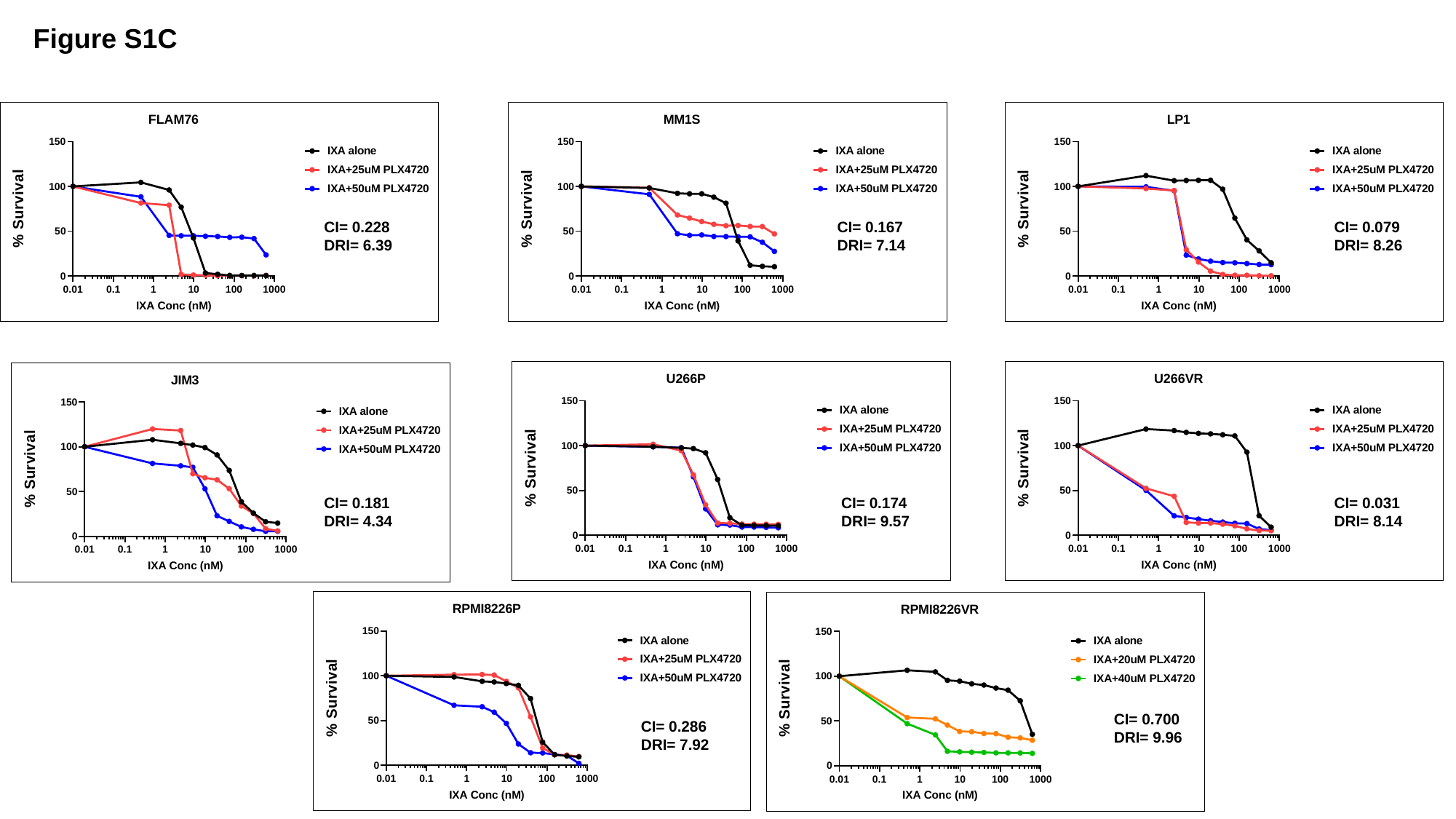

Figure S1C
CI= 0.228
DRI= 6.39
CI= 0.167
DRI= 7.14
CI= 0.079
DRI= 8.26
CI= 0.181
DRI= 4.34
CI= 0.174
DRI= 9.57
CI= 0.031
DRI= 8.14
CI= 0.700
DRI= 9.96
CI= 0.286
DRI= 7.92

## Slide 5
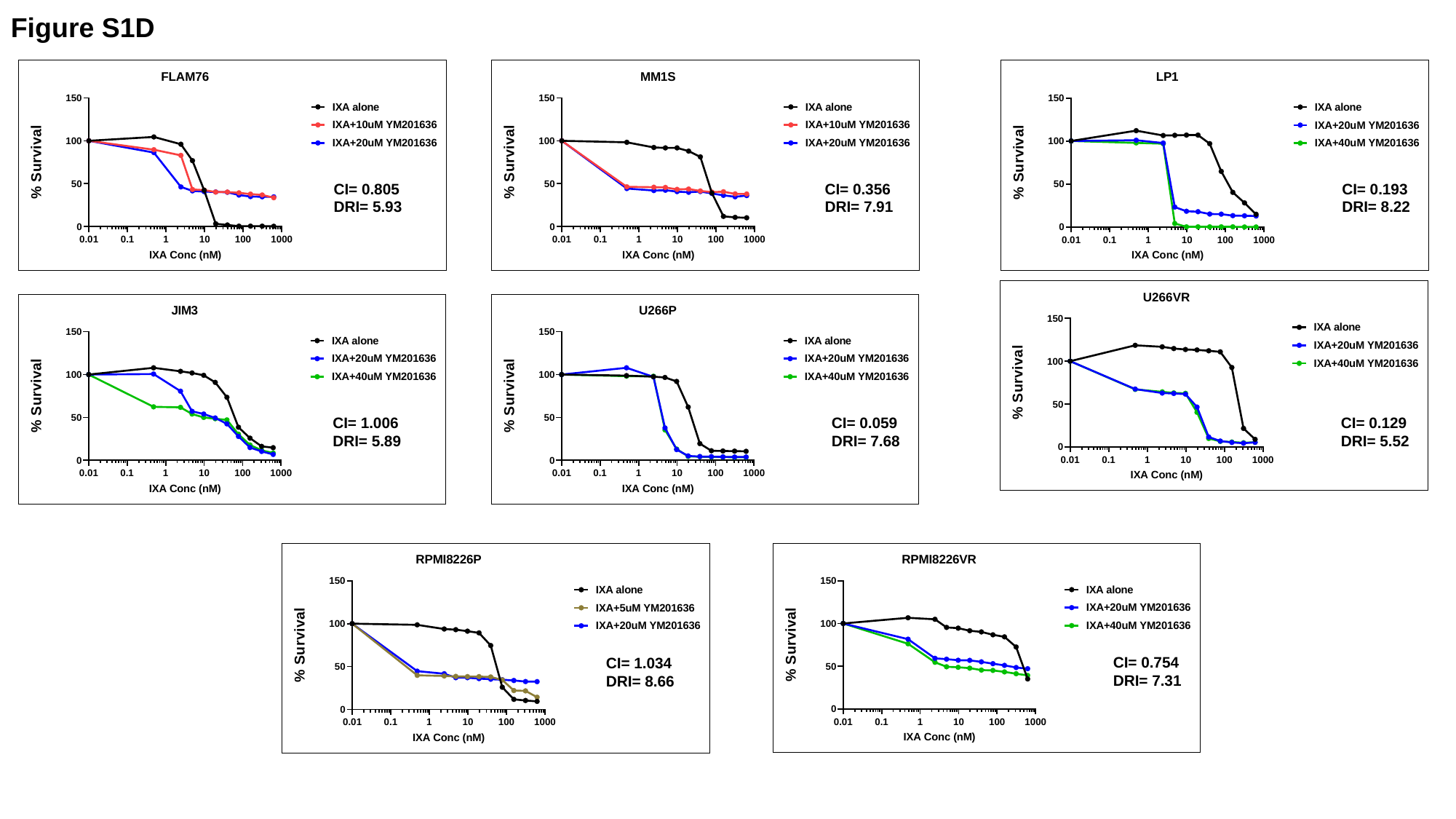

Figure S1D
CI= 0.805
DRI= 5.93
CI= 0.356
DRI= 7.91
CI= 0.193
DRI= 8.22
CI= 1.006
DRI= 5.89
CI= 0.059
DRI= 7.68
CI= 0.129
DRI= 5.52
CI= 0.754
DRI= 7.31
CI= 1.034
DRI= 8.66

## Slide 6
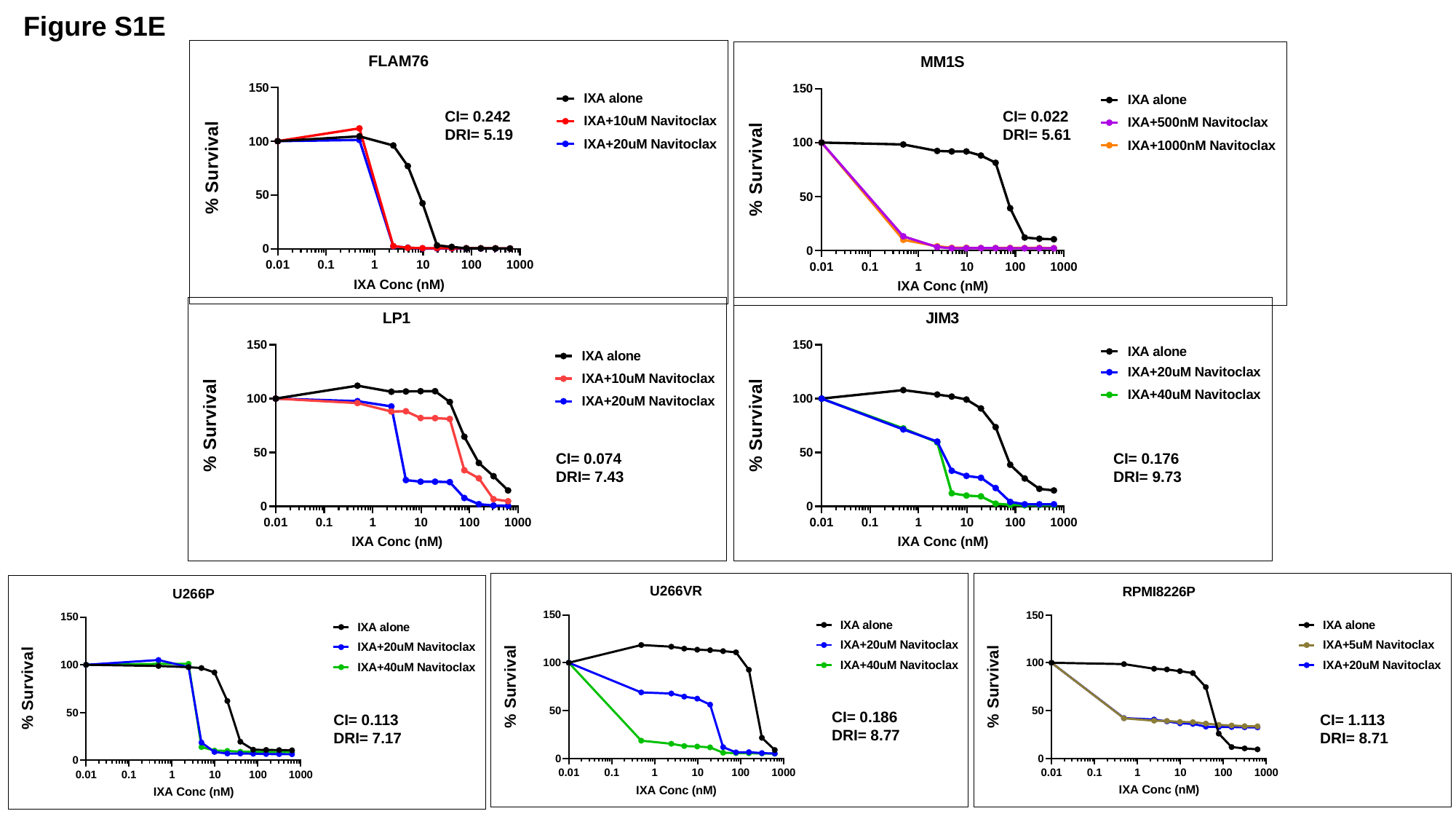

Figure S1E
CI= 0.242
DRI= 5.19
CI= 0.022
DRI= 5.61
CI= 0.074
DRI= 7.43
CI= 0.176
DRI= 9.73
CI= 0.186
DRI= 8.77
CI= 0.113
DRI= 7.17
CI= 1.113
DRI= 8.71

## Slide 7
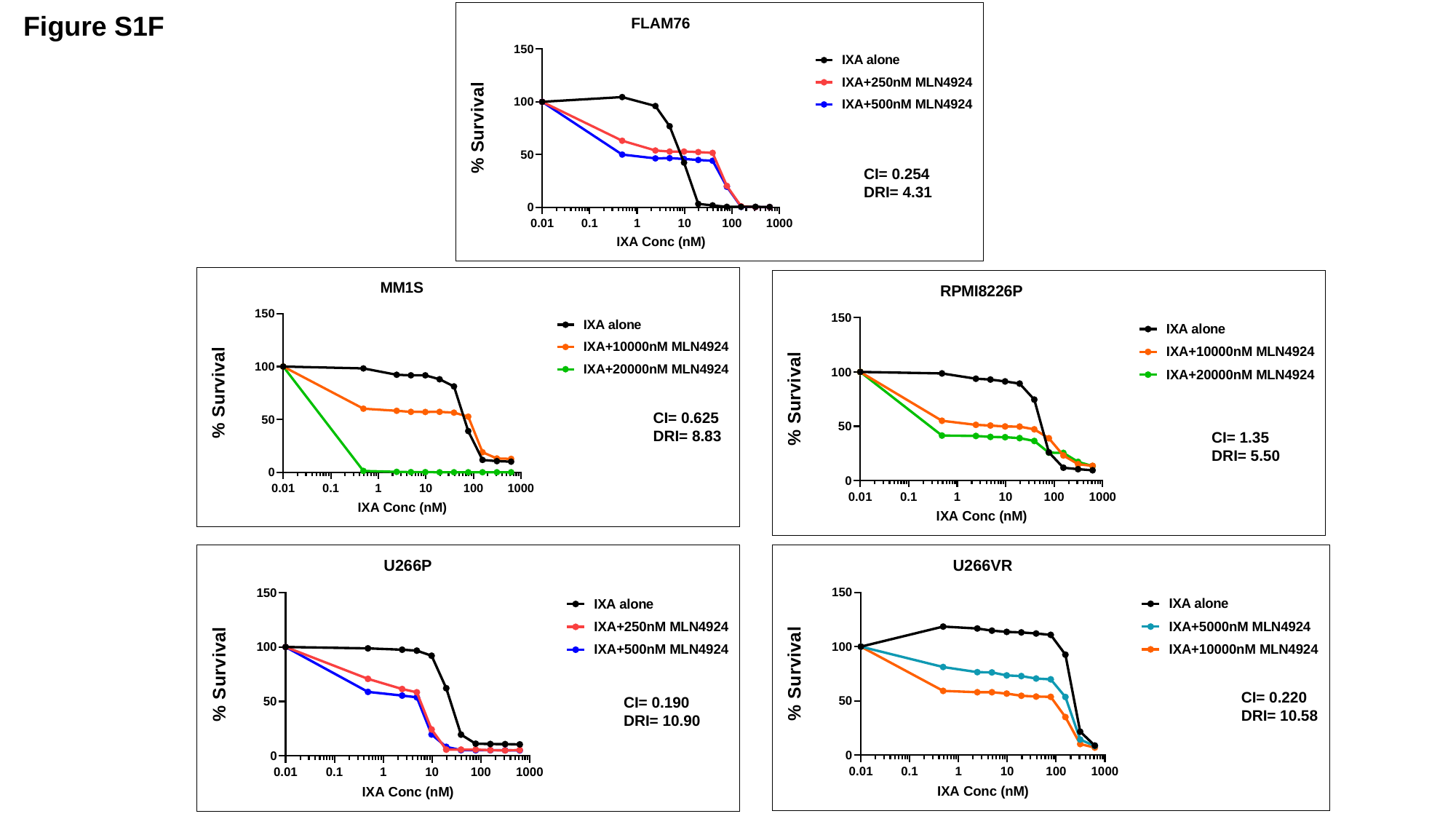

Figure S1F
CI= 0.254
DRI= 4.31
CI= 0.625
DRI= 8.83
CI= 1.35
DRI= 5.50
CI= 0.220
DRI= 10.58
CI= 0.190
DRI= 10.90

## Slide 8
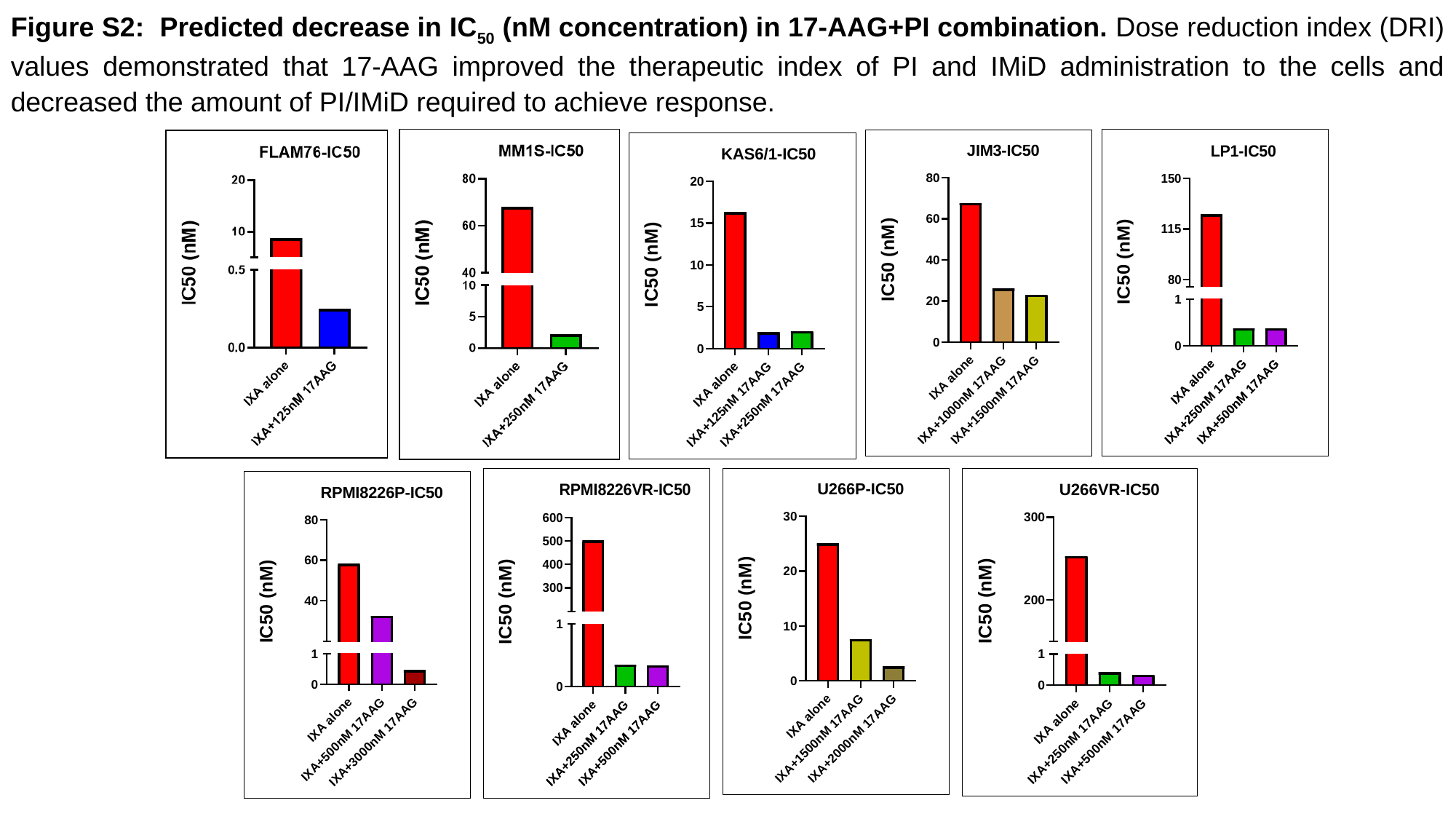

Figure S2: Predicted decrease in IC50 (nM concentration) in 17-AAG+PI combination. Dose reduction index (DRI) values demonstrated that 17-AAG improved the therapeutic index of PI and IMiD administration to the cells and decreased the amount of PI/IMiD required to achieve response.

## Slide 9
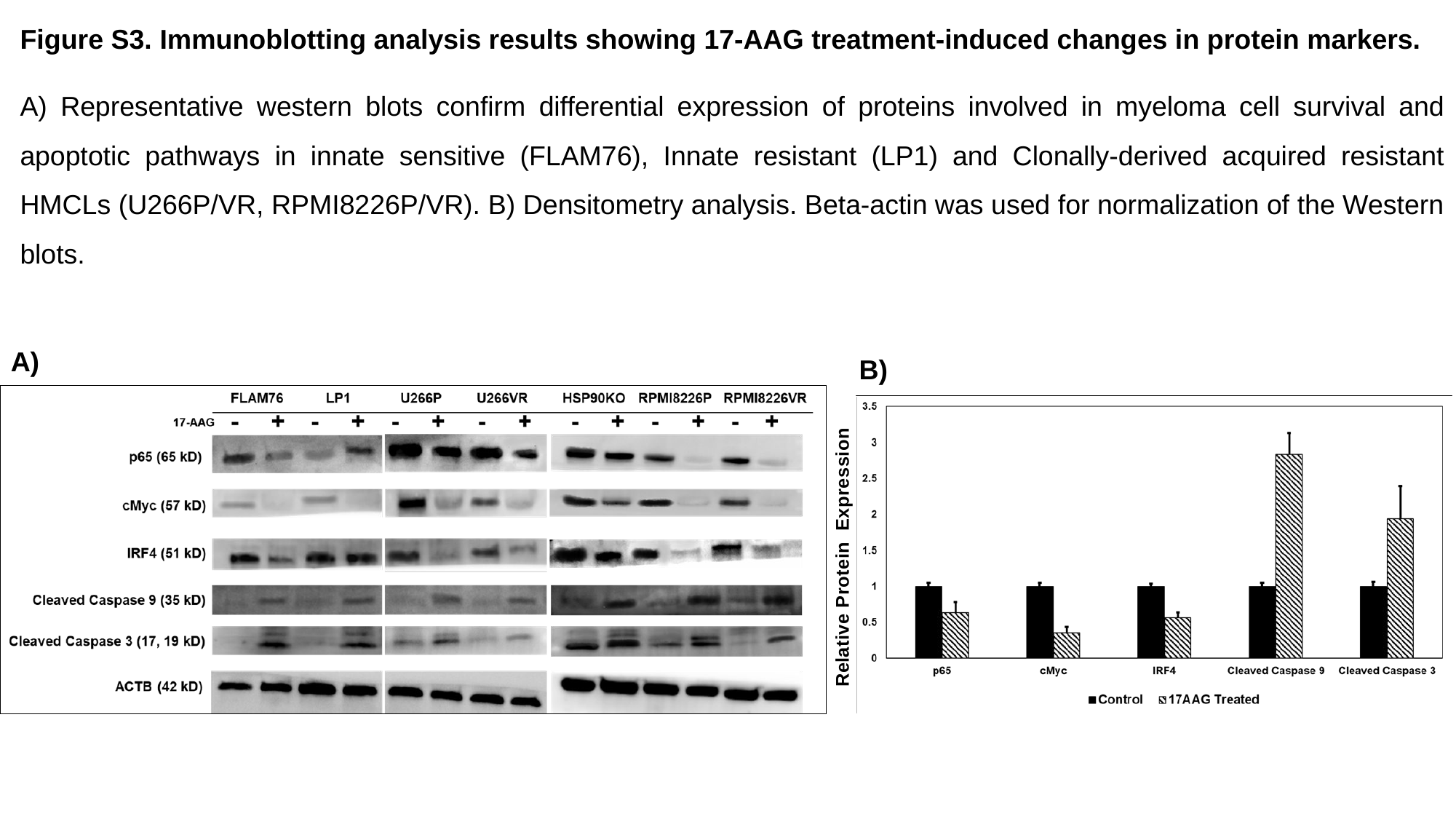

Figure S3. Immunoblotting analysis results showing 17-AAG treatment-induced changes in protein markers.
A) Representative western blots confirm differential expression of proteins involved in myeloma cell survival and apoptotic pathways in innate sensitive (FLAM76), Innate resistant (LP1) and Clonally-derived acquired resistant HMCLs (U266P/VR, RPMI8226P/VR). B) Densitometry analysis. Beta-actin was used for normalization of the Western blots.
A)
B)

## Slide 10
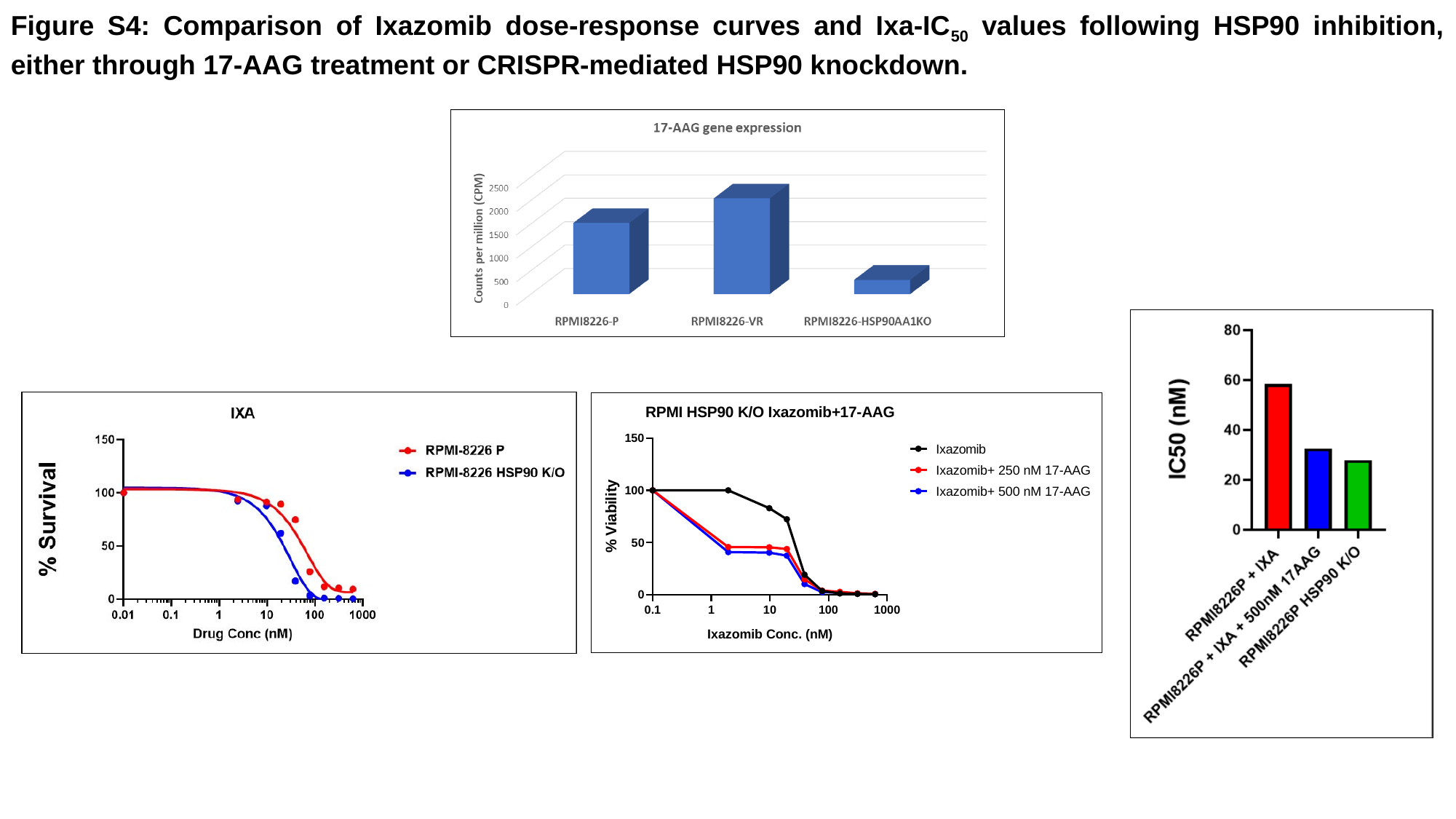

Figure S4: Comparison of Ixazomib dose-response curves and Ixa-IC50 values following HSP90 inhibition, either through 17-AAG treatment or CRISPR-mediated HSP90 knockdown.

## Slide 11
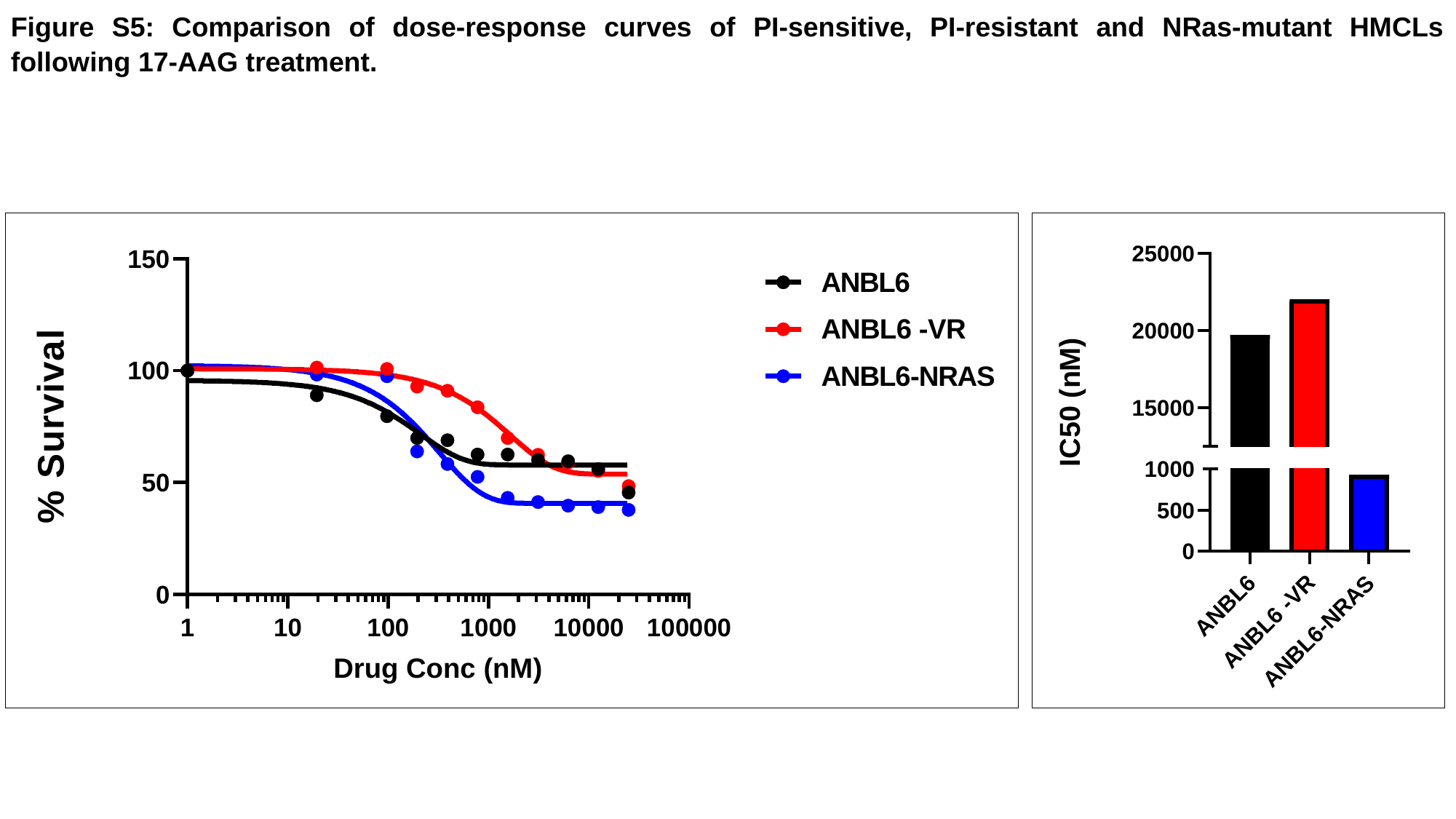

Figure S5: Comparison of dose-response curves of PI-sensitive, PI-resistant and NRas-mutant HMCLs following 17-AAG treatment.

## Slide 12
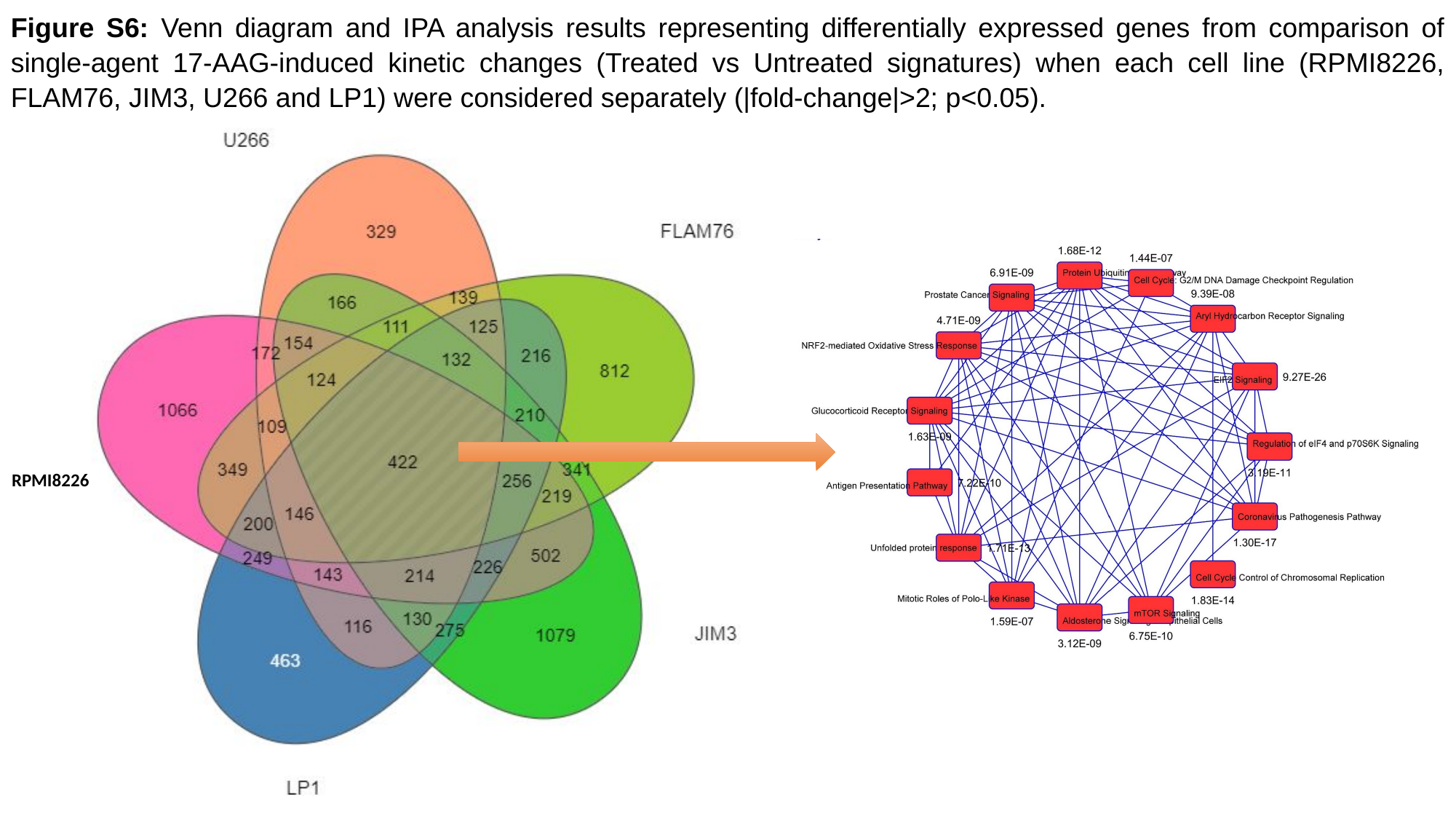

Figure S6: Venn diagram and IPA analysis results representing differentially expressed genes from comparison of single-agent 17-AAG-induced kinetic changes (Treated vs Untreated signatures) when each cell line (RPMI8226, FLAM76, JIM3, U266 and LP1) were considered separately (|fold-change|>2; p<0.05).
RPMI8226

## Slide 13
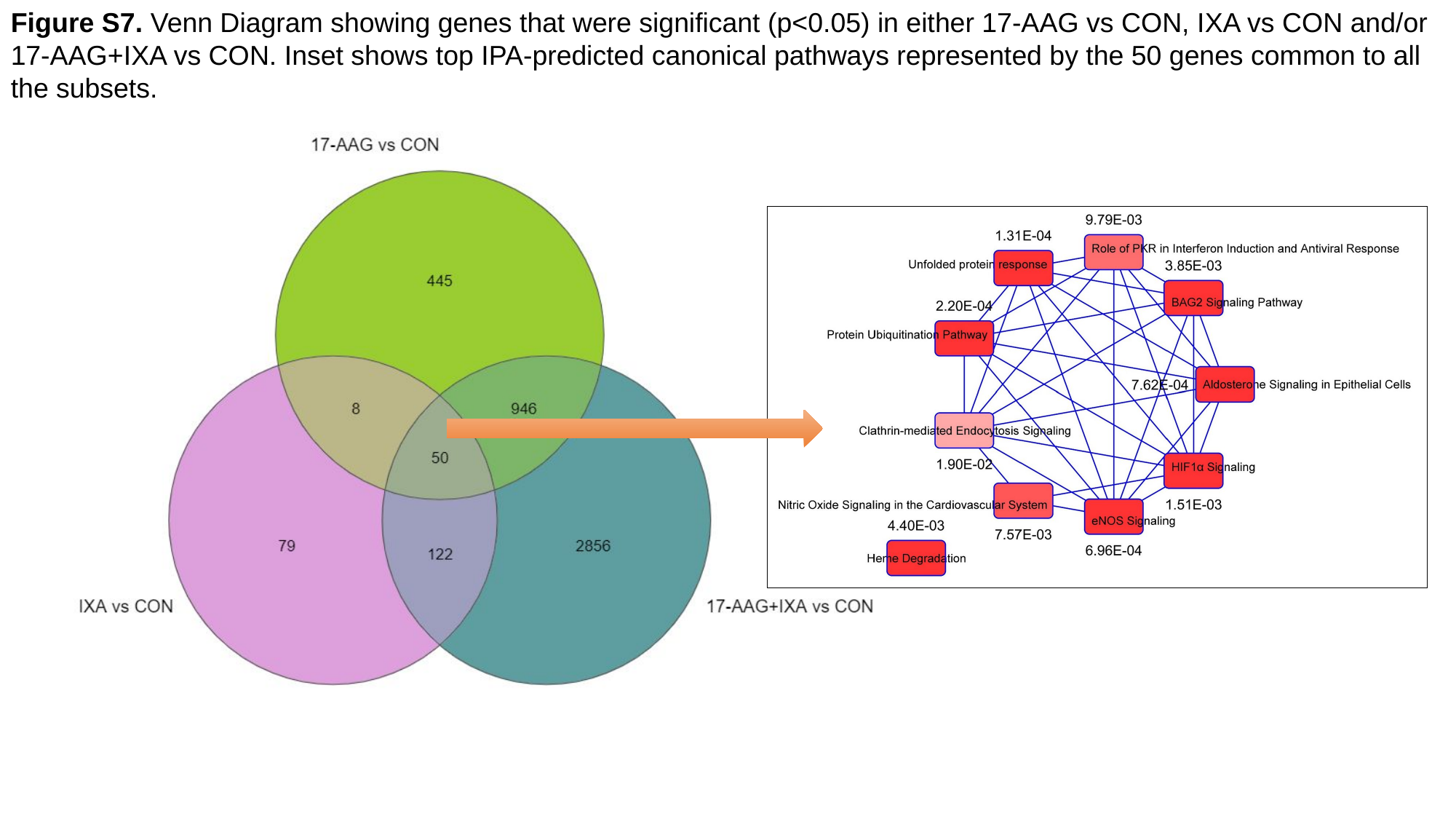

Figure S7. Venn Diagram showing genes that were significant (p<0.05) in either 17-AAG vs CON, IXA vs CON and/or 17-AAG+IXA vs CON. Inset shows top IPA-predicted canonical pathways represented by the 50 genes common to all the subsets.

## Slide 14
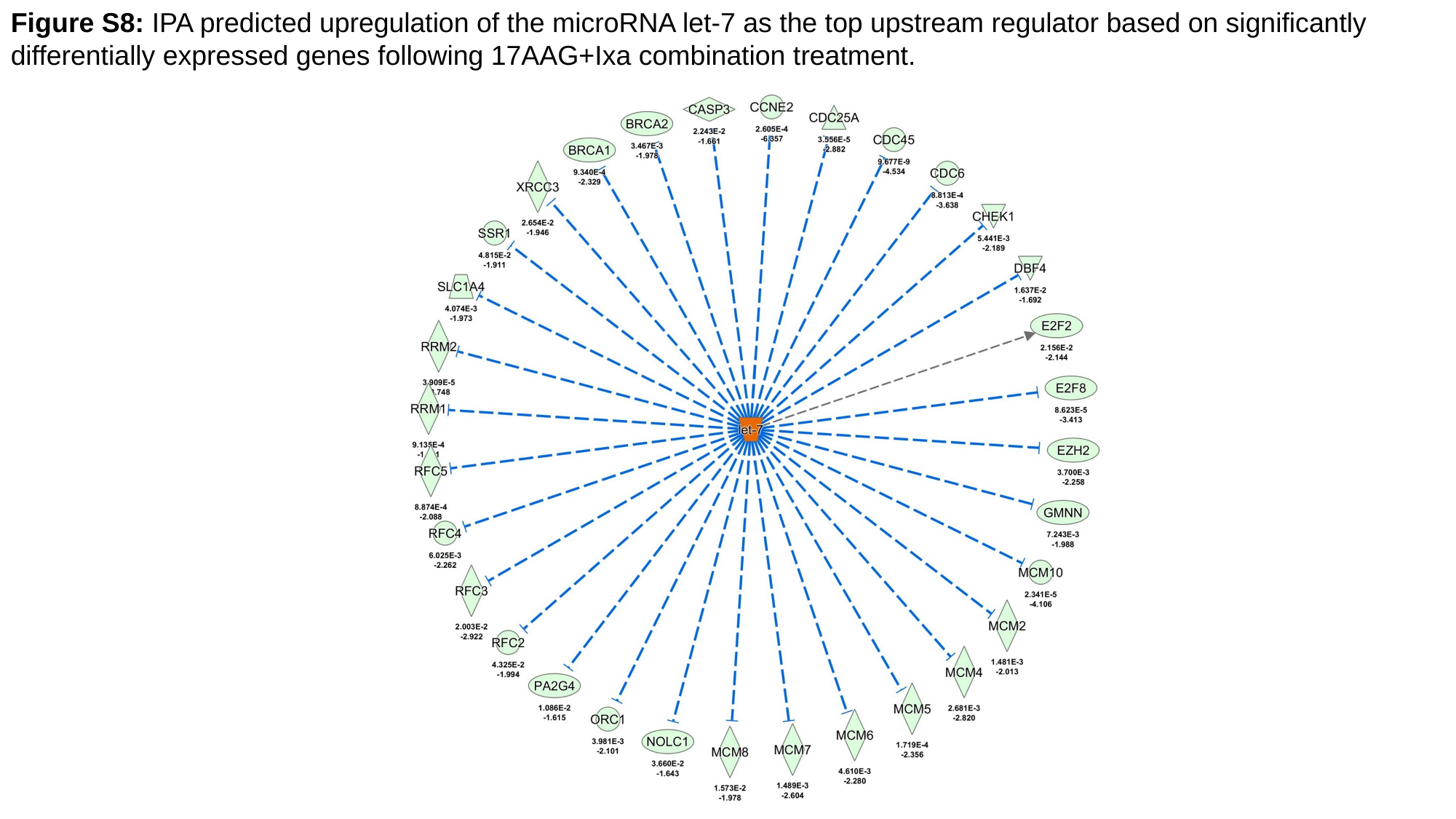

Figure S8: IPA predicted upregulation of the microRNA let-7 as the top upstream regulator based on significantly differentially expressed genes following 17AAG+Ixa combination treatment.
